# Global gene expression of histologically normal primary skin cells from BCNS subjects reveals “single-hit” effects that are influenced by rapamycin

**DOI:** 10.18632/oncotarget.26640

**Published:** 2019-02-15

**Authors:** Amruta Phatak, Mohammad Athar, James A. Crowell, David Leffel, Brittney-Shea Herbert, Allen E. Bale, Levy Kopelovich

**Affiliations:** ^1^ Department of Medical and Molecular Genetics, Indiana University School of Medicine, Indianapolis, IN, USA; ^2^ Department of Dermatology, University of Alabama at Birmingham, Birmingham, AL, USA; ^3^ NCI-DCTD-DTP, Bethesda, MD, USA; ^4^ Department of Dermatology, Yale School of Medicine, New Haven, CT, USA; ^5^ Department of Genetics, Yale School of Medicine, New Haven, CT, USA; ^6^ Department of Medicine, Weill Cornell Medical College, New York, NY, USA

**Keywords:** Gorlin syndrome, basal cell carcinoma, patched, HH signaling, rapamycin

## Abstract

Studies of dominantly heritable cancers enabled insights about tumor progression. BCNS is a dominantly inherited disorder that is characterized by developmental abnormalities and postnatal neoplasms, principally BCCs. We performed an exploratory gene expression profiling of primary cell cultures derived from clinically unaffected skin biopsies of BCNS gene-carriers (*PTCH1* ^+/-^) and normal individuals. PCA and HC of untreated keratinocytes or fibroblasts failed to clearly distinguish BCNS samples from controls. These results are presumably due to the common suppression of canonical HH signaling *in vitro*. We then used a relaxed threshold (p-value <0.05, no FDR cut-off; FC 1.3) that identified a total of 585 and 857 genes differentially expressed in BCNS keratinocytes and fibroblasts samples, respectively. A GSEA identified pancreatic β cell hallmark and mTOR signaling genes in BCNS keratinocytes, whereas analyses of BCNS fibroblasts identified gene signatures regulating pluripotency of stem cells, including WNT pathway. Significantly, rapamycin treatment (FDR<0.05), affected a total of 1411 and 4959 genes in BCNS keratinocytes and BCNS fibroblasts, respectively. In contrast, rapamycin treatment affected a total of 3214 and 4797 genes in normal keratinocytes and normal fibroblasts, respectively. The differential response of BCNS cells to rapamycin involved 599 and 1463 unique probe sets in keratinocytes and fibroblasts, respectively. An IPA of these genes in the presence of rapamycin pointed to hepatic fibrosis/stellate cell activation, and HIPPO signaling in BCNS keratinocytes, whereas mitochondrial dysfunction and *AGRN* expression were uniquely enriched in BCNS fibroblasts. The gene expression changes seen here are likely involved in the etiology of BCCs and they may represent biomarkers/targets for early intervention.

## INTRODUCTION

Basal cell carcinomas (BCCs) of the skin represent nearly one half of all cancers diagnosed in the United States [1, 2]. Although these tumors rarely metastasize and are an infrequent cause of cancer mortality [3], they are associated with significant morbidity due to local invasion and tissue destruction. The basal cell nevus syndrome (BCNS), also known as Gorlin syndrome or the nevoid basal cell carcinoma syndrome, is an autosomal dominant disorder characterized by multiple BCCs, occasional childhood malignancies, and developmental defects including palmar pits, jaw cysts, coarse facial appearance, malformations of the ribs, spine and brain, macrocephaly, and generalized overgrowth [4–6].

The majority of BCNS cases can be attributed to heterozygous germline mutations in *PTCH1*; the human homolog of the Drosophila gene, *patched* [7]. *Patched* (*PTCH1*) encodes the receptor for hedgehog (HH), a secreted morphogen involved in establishing the basic framework of developing embryos in Drosophila and in other organisms, including vertebrates [8, 9]. In the canonical pathway, binding of HH to *PTCH* releases inhibition on SMO, which then signals through a series of steps to zinc finger trans-activators that ultimately convey the signal to the nucleus. In humans, the trans-activator function is performed by *GLI1*, *GLI2* and *GLI3*. Genes directly regulated by GLI include *PTCH1*, *HIP* (hedgehog interacting protein), and *GLI1* itself, in many tissues. A host of other potential target genes in normal cells have been identified by expression microarrays where it appears that HH signaling is influenced by the stage of development and tissue-specific factors [8, 9], leading to different global expression patterns in different organs [10].

Appropriately, tumor suppressor genes and their effector pathways have been identified in several dominantly heritable cancers, providing insights about cancer initiation and potential intervention [11–13]. Analogous to retinoblastoma [11, 12] and other autosomal dominant forms of heritable cancer [13–18], BCCs in BCNS patients, arise through a two-hit mechanism in which “one hit” is an inherited, inactivating mutation in *PTCH1*, and the second hit is a somatically derived mutation in the remaining *PTCH1* allele [7]. The great majority of sporadic BCCs have two somatically derived mutations in *PTCH1*, or less often, an activating mutation in *SMO* that mimics loss of *PTCH1* [7, 19–23]. Although additional molecular changes are probably necessary for the development of BCCs, it is likely that loss of *PTCH1* function and consequent activation of the hedgehog pathway is a necessary early step [24, 25].

This “one-hit model” for BCNS is supported by many features of this disorder such as developmental defects that have a generalized or symmetric manifestation which would be difficult to reconcile with a two-hit mechanism. Generalized overgrowth, acromegalic bone structure, and relative macrocephaly, for example, suggest that heterozygous loss of function leads to cellular hyper-proliferation in bone or cartilage [26]. In this regard, expression analysis of BCNS fibroblasts and fibroblasts from unaffected individuals [27] implicated genes that were hypothesized to contribute to a growth advantage in BCNS fibroblasts.

BCNS patients can develop thousands of skin tumors over their lifetimes, and treating this multiplicity of tumors with conventional surgical or other ablative therapies is a challenge. Preventive or curative medical therapy with an effective targeted agent that is known to have limited adverse, off-target effects would represent a great advance. Although several compounds are known to inhibit hedgehog signaling [28–30], their overall preventative efficacy of BCC remains to be established, and side effects limit their long-term use. In this regard, it is important to note that cell identity switch allows residual BCC to survive hedgehog pathway inhibition, necessitating the use of an additional agent(s) [31].

Rapamycin, also known as sirolimus, is an antibiotic isolated from Streptomyces hygroscopicus [32] that blocks mitogenic signaling by growth factor receptors and is a promising anti-neoplastic agent for a variety of tumors [33–37]. The best studied pathway affected by rapamycin is that conducted by the mTOR complex 1 (mTORC1), which consists of the mTOR protein (a member of the (PI(3)K) family) DEPTOR, PRAS40, RAPTOR, mLST8, and TTI1–TEL2 [33–37]. Importantly, rapamycin has also been shown to have an effect on the canonical HH pathway wherein crosstalk between mTOR and HH pathways has been indicated [38–40]. Thus, while blocking mTOR may take away a signaling component that is necessary but not sufficient for transformation, the mTOR and HH pathways coincidentally may target an overlapping set of genes that are likely to inhibit BCC carcinogenesis. Incidentally, rapamycin has been shown to inhibit BCC development in kidney transplant patients [41, 42] and has also been shown in a case report to inhibit BCCs, advocating its use when surgical intervention is counter-indicated [43].

The first aim of this study was to profile baseline global gene expression in cells obtained from histologically normal skin of BCNS subjects and compare these with skin cells from unaffected individuals to detect “single-hit” effects caused by *PTCH1* (+/-). Since hedgehog activation has been proposed to function in carcinogenesis through paracrine activity on stroma as well as cell autonomous activity [44, 45], both keratinocytes and fibroblasts were analyzed in this study. Our second aim was to determine if a particular set of genes was differentially influenced by rapamycin in tissues that might have been “sensitized” to its effect by loss of one copy of *PTCH1*.

## RESULTS

### Exploratory analysis at baseline reveals no clear association of samples based on *PTCH1* mutation status

**Figure 1:**
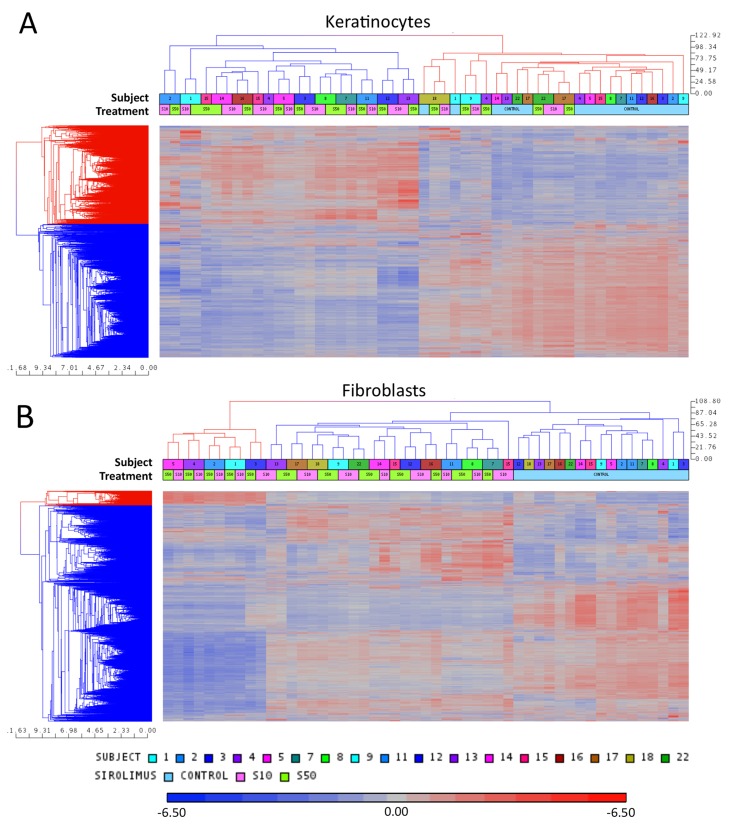
Unsupervised hierarchical clustering of microarray data of keratinocytes **(A)** and fibroblasts **(B)** for each subject with or without (control) rapamycin treatment. Coefficient of variation of more than 0.1. The subject numbers match the participant ID numbers in Table 1. S10 indicates sirolimus/rapamycin low dose and S50 indicates sirolimus/rapamycin high dose.

The first goal of this study was to explore changes in gene expression that can be attributed to mutations in *PTCH1* and can possibly contribute to molecular abnormalities of basal cell carcinomas. Here we profiled gene expression patterns of *PTCH1* (+/-) keratinocytes and *PTCH1* (+/-) fibroblasts derived from histologically normal tissues of BCNS subjects and compared them to cells derived from normal individuals at baseline and following treatment with rapamycin (Table 1). A principal components analysis (PCA) (Supplementary Figure 1) revealed that although the two treatments clustered separately, i.e., with and without rapamycin, the differences in gene expression due to *PTCH1* mutation did not result in a significant clustering and separation of BCNS from normal samples, indicating that the *PTCH1* mutation itself, whether truncating or missense, does not alter global gene expression of keratinocytes or fibroblasts enough to provide clear separation between BCNS and normal specimens. The lack of clear delineation of samples at baseline can be attributed to an attenuated canonical HH pathway in *PTCH1* (+/-) cells grown in culture [46, 47]. However, rapamycin treatment caused significant changes in global gene expression pattern that provided a clear separation of treated and untreated samples derived from either BCNS patients or normal subjects (Figure 1).

**Table 1:** Characteristics of cases and controls

Participant ID	Gender	Age		PTCH1 Sequencing	Mutation Type	Clinical features		
						BCCs	Jaw Cysts	Pits
**Cases**								
04	F	55		W278X	Truncating	+	+	+
11	F	60		W844G^1^	Missense	+	+	+
12	F	51		W926L^2^	Missense	+	+	+
14	F	30	Relative of 15, 16	Q853X^3^	Truncating	+	+	+
07	M	20		Codon 1124delC	Truncating	+	+	+
13	M	25	Relative of 12	W926L^2^	Missense	+	+	+
17	M	44		C1043X	Truncating	+	+	+
18	M	46		IVS15+9 G>C^4^	Missense	+	+	+
22	M	50		Exon 1E splice acceptor^5^	Missense	+	+	-
**Controls**								
02	F	41		Not done	-	-	-	-
08	F	21	Relative of 07	Wild type	-	-	-	-
09	F	27		Not done	-	-	-	-
15	F	54	Relative of 14	Wild type	-	-	-	-
01	M	37		Not done	-	-	-	-
03	M	51		Not done	-	-	-	-
05	M	49	Relative of 04	Wild type	-	-	-	-
16	M	53	Relative of 14	Wild type	-	-	-	-

^1^Trp at amino acid 844 is 100% conserved in vertebrates and invertebrates. Codon numbering is according to NP_000255.

^2^Trp at amino acid 926 is 100% conserved in vertebrates and invertebrates; W926R reported previously in BCNS [67].

^3^Truncating mutation has been reported [67].

^4^Variant of unknown significance; however, *PTCH1* mutations p.W926X/R have been reported in 5 cases [68].

^5^Creates an abnormal acceptor site predicted to be highly efficient at position 32 of exon 1E (G to A change).

The PCA results were further supported by unsupervised hierarchical clustering of genes (coefficient of variation of more than 0.1 in the microarray data) at baseline and after rapamycin treatment (Figure 1). While control samples distinctly cluster apart from rapamycin treated samples for both the keratinocytes (Figure 1A) and fibroblasts (Figure 1B), the clustering pattern was independent of *PTCH1* mutation status or rapamycin concentration. Together, these results reaffirm our observation that mono-allelic *PTCH1* mutation did not affect the global gene expression pattern to distinguish BCNS from normal samples *in vitro*. Secondly, the analysis suggests that the effect of rapamycin at a more stringent level of statistical scrutiny (FDR<0.05) is dependent on the intrinsic gene expression pattern unique to the sample derived from each individual.

### Differentially regulated genes in BCNS and normal samples at baseline under more relaxed statistical constrains

Following 5-way mixed-model ANOVA-REML (FC >1.3 and <-1.3; unadjusted p<0.05), we found several gene sets that were differentially expressed. Briefly, the baseline comparison of microarray data of keratinocytes and fibroblasts showed 585 and 857, respectively of differentially expressed genes between BCNS and normal subjects. Hence, relaxing the strict FDR cut-off, effected the unmasking of candidate genes that are differentially expressed at baseline in this hypothesis-generating analysis. The top up-regulated genes at baseline in keratinocytes derived from BCNS patients were *NFIA-AS2*, *RBMY3AP*, *SEC16B*, *CROCC*, *SYN1*, *MKL1*, *FAM71A*, *LOC101928973*, *SIM2*, *GNG8* in comparison to normal keratinocytes, whereas *GSTT1*, *STEAP4*, *NHLH2*, *MAST4*, *KLHL24*, *MALAT1*, *ARRDC3*, *LOC101928100*, *PELI*, *SLC28A3* were the most down-regulated genes (Table 2). The top up-regulated genes at baseline in BCNS fibroblasts were *TMEM155*, *ABAT*, *BEX1*, *PDE4DIP*, *CPM*, *HNMT*, *SFRP2*, *AKR1C3*, *RAB27B*, *PLAC8,* whereas *GOLGA8A/GOLGA8B*, *NEAT1*, *MALAT1*, *MEG3*, *COL4A1*, *BCAT1*, *E2F7*, *FN1*, *LOC100190986*, *HELLS* were the most down-regulated (Table 2). The ANOVA design and the comprehensive gene lists used for baseline comparison are provided in Supplementary File 1.

**Table 2:** List of top genes differentially regulated in keratinocytes and fibroblasts derived from BCNS subjects compared to normal individuals

Gene Symbol	Gene title (HGNC approved)	Fold change	p-value
***Genes up-regulated in BCNS keratinocytes***			
NFIA-AS2	Nuclear factor I/A antisense RNA 2	1.67846	0.000241
RBMY3AP	RNA binding motif protein, Y-linked, family 3, member A pseudogene	1.61282	0.000475
SEC16B	SEC16 homolog B (S. cerevisiae)	1.59121	9.05E-05
CROCC	ciliary rootlet coiled-coil, rootletin	1.58479	0.000707
SYN1	synapsin I	1.54555	0.000235
MKL1	Megakaryoblastic leukemia (translocation) 1	1.54463	0.004594
FAM71A	family with sequence similarity 71, member A	1.53406	0.001157
LOC101928973	uncharacterized	1.53286	0.000580
SIM2	single-minded homolog 2 (Drosophila)	1.52543	0.000744
GNG8	guanine nucleotide binding protein (G protein), gamma 8	1.52347	0.001798
***Genes down-regulated in BCNS keratinocytes***			
GSTT1	glutathione S-transferase theta 1	-2.84883	0.014633
STEAP4	STEAP family member 4	-2.51858	0.011631
NHLH2	nescient helix loop helix 2	-2.21331	0.001196
MAST4	Microtubule Associated Serine/Threonine Kinase Family Member 4	-2.06517	0.016778
KLHL24	kelch-like 24 (Drosophila)	-2.05221	0.010495
MALAT1	metastasis associated lung adenocarcinoma transcript 1 (non-protein coding)	-1.95113	0.033836
ARRDC3	arrestin domain containing 3	-1.84582	0.032566
LOC101928100	uncharacterized	-1.83053	0.013417
PELI1	Pellino homolog 1 (Drosophila)	-1.77824	0.045639
SLC28A3	solute carrier family 28 (sodium-coupled nucleoside transporter), member 3	-1.77746	0.035360
***Genes up-regulated in BCNS fibroblasts***			
TMEM155	transmembrane protein 155	2.61437	0.008088
ABAT	4-aminobutyrate aminotransferase	2.55331	0.011422
BEX1	brain expressed, X-linked 1	2.3517	0.027068
PDE4DIP	phosphodiesterase 4D interacting protein	2.32946	0.035918
CPM	carboxypeptidase M	2.27972	0.027836
HNMT	histamine N-methyltransferase	2.25939	0.035685
SFRP2	secreted frizzled-related protein 2	2.25661	0.036160
AKR1C3	aldo-keto reductase family 1, member C3 (3-alpha hydroxysteroid dehydrogenase, type II)	2.23228	0.046084
RAB27B	RAB27B, member RAS oncogene family	2.14117	0.004020
PLAC8	placenta-specific 8	2.13321	0.011749
***Genes down-regulated in BCNS fibroblasts***			
GOLGA8A	golgin A8 family, member A	-2.88122	0.013547
NEAT1	Nuclear Paraspeckle assembly transcript 1 (non-protein coding)	-2.37719	0.032147
MALAT1	metastasis associated lung adenocarcinoma transcript 1 (non-protein coding)	-2.36124	0.023835
MEG3	maternally expressed 3 (non-protein coding)	-2.34455	0.019488
COL4A1	collagen, type IV, alpha 1	-2.26454	0.022223
BCAT1	branched chain amino-acid transaminase 1, cytosolic	-2.16931	0.018688
E2F7	E2F transcription factor 7	-2.16325	0.029640
FN1	fibronectin 1	-2.1232	0.027185
LOC100190986	hypothetical LOC100190986	-2.09273	0.022763
HELLS	helicase, lymphoid-specific	-2.08964	0.009022

### GSEA specifies baseline differences between BCNS and normal keratinocytes or fibroblasts

**Figure 2:**
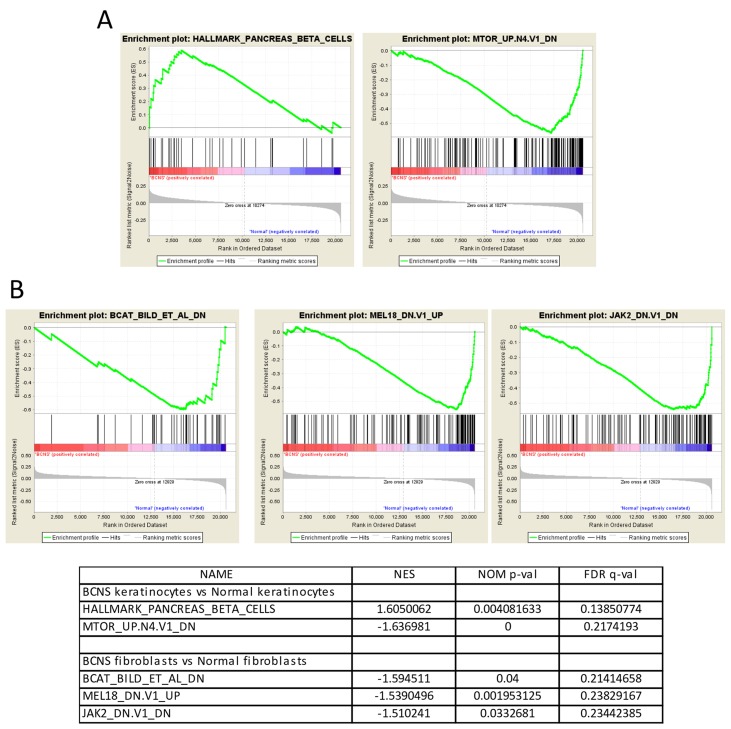
Gene set enrichment analyses (GSEA) on BCNS keratinocytes and fibroblasts compared to normal samples **(A)** GSEA shows that the ‘HALLMARK_PANCREAS_BETA_CELLS’ gene-set comprising of genes specifically up-regulated in pancreatic beta cells is enriched in BCNS keratinocytes as compared to the normal keratinocyte samples. The GSEA analysis also indicates that the ‘MTOR_UP.N4.V1_DN’ gene set containing down-regulated genes post rapamycin treatment is enriched in the normal keratinocytes **(A)**. **(B)** For the fibroblasts, GSEA shows that the ‘BCAT_BILD_ET_AL_DN’ gene set comprising of genes down-regulated due to activated CTNNB1 overexpression; the ‘MEL18_DN.V1_UP’ gene set comprising of up-regulated genes in medullablastoma cells after PCGF2 knockdown; and the ‘JAK2_DN.V1_DN’ gene set containing the genes down-regulated by JAK2 knock-down are all enriched in the normal fibroblast samples. For the enrichment plots, profile of running ES score and position of GeneSet members on the rank ordered list are denoted for HALLMARK_PANCREAS_BETA_CELLS **(A)**, MTOR_UP.N4.V1_DN **(A)**, BCAT_BILD_ET_AL_DN **(B)**, MEL18_DN.V1_UP **(B)**, JAK2_DN.V1_DN **(B)**, and the respective normalized enrichment scores (NES), nominal p-values (NOM p-val), and q-values (FDR q-val) are summarized in table below the plots.

Next, we performed baseline gene set enrichment analyses (GSEA) of both keratinocytes and fibroblasts samples derived from BCNS patients and compared them to the control group (Figure 2). The results indicated a positive correlation of BCNS keratinocytes with predefined pancreatic β cell hallmark gene set that comprises 40 genes, specifically upregulated in pancreatic beta cells and known to be involved in glucose metabolism and dysglycemia (Figure 2A). Interestingly, BCNS keratinocytes, in contrast to normal keratinocytes, correlated negatively with the oncogenic gene signature mTOR_UP.N4.V1_DN in the MSigDb (Figure 2A and Supplementary File 2), comprising genes shown to be down-regulated upon treatment with rapamycin [48]. In the case of BCNS fibroblasts, compared with normal fibroblasts, GSEA pointed to a negative correlation with three oncogenic signatures in the MsigDb that comprised genes down-regulated in response to activated CTNNB1 overexpression (BCAT_BILD_ET_AL_DN) and RNAi-mediated JAK2 knockdown (JAK2_DN.V1_DN), and genes up-regulated upon RNAi-mediated knockdown of PCGF2 (MEL18_DN.V1_UP) (Figure 2B).

### Biological differences attributed to rapamycin effects in BCNS and normal keratinocytes

**Figure 3:**
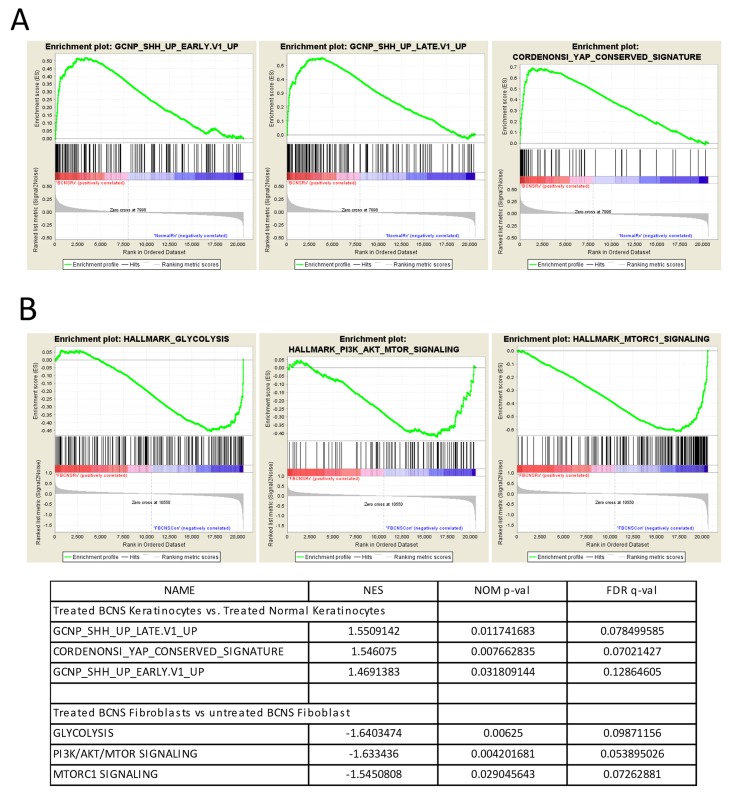
Gene set enrichment analyses (GSEA) on rapamycin-treated BCNS keratinocytes and fibroblasts compared to normal samples **(A)** GSEA shows that the ‘GCNP_SHH_UP_EARLY.V1_UP’ gene set consisting of genes up-regulated in granule cell neuron precursors (GCNP) after Shh stimulation for 3h are positively correlated and enriched in BCNS keratinocytes treated with rapamycin. Interestingly, GSEA also indicates the enrichment of a YAP conserved signature in the rapamycin treated BCNS keratinocytes. **(B)** For the fibroblasts, GSEA indicates that normal fibroblasts treated with rapamycin correlate with the hallmark gene sets namely ‘GLYCOLYSIS’, ‘PI3K/AKT/MTOR SIGNALING’, and ‘MTORC1 SIGNALING’ as compared to rapamycin treated BCNS fibroblasts. For the enrichment plots, profile of running ES score and position of GeneSet members on the rank ordered list for are denoted for oncogenic signatures ‘GCNP_SHH_UP_LATE.V1_UP’ **(A)**, CORDENONSI_YAP_CONSERVED_SIGNATURE’ **(A)**, ‘GCNP_SHH_UP_EARLY.V1_UP’ **(A)**, and the hallmark gene sets namely ‘GLYCOLYSIS’ **(B)**, PI3K/AKT/MTOR SIGNALING **(B)**, MTORC1 SIGNALING **(B)** and the respective normalized enrichment scores (NES), nominal p-values (NOM p-val), and q-values (FDR q-val) are summarized in table below the plots.

Here we used GSEA algorithm on comprehensive microarray dataset without preprocessing to determine an *a priori* defined gene sets that exhibit statistically significant and biologically relevant differences between untreated and rapamycin treated BCNS keratinocytes and fibroblasts. The GSEA results indicated several significant pathways that were altered by rapamycin treatment in both BCNS keratinocytes and BCNS fibroblasts (Figure 3, Supplementary Files 2–4).

In BCNS keratinocytes, enriched signatures in the presence of rapamycin signal the involvement of and regulation by MYC and G2/M checkpoint genes, cell cycle related targets of E2F transcription factor and mitotic spindle assembly [49]. The oncogenic signatures enriched in BCNS keratinocyte treated with rapamycin showed sets of genes up-regulated in neuronal precursors after Shh stimulation, PIGF treatment, genes governed by overexpression of E2F1 and E2F3, and genes up-regulated in primary keratinocytes from RB1 and RBL1 skin-specific knockout mice. Interestingly as well, the YAP conserved signature [49–51], was also shown to be concordant with rapamycin treated BCNS keratinocytes (Figure 3A, Supplementary Files 2–4). The oncogenic signatures comprising of down-regulated genes leading to CTNNB1 overexpression, SRC overexpression, oncogenic KRAS expression in context of TBK1 knockdown, RNAi mediated knockdown of genes such as EIF4G1, HOXA9 and RPS14, and genes down-regulated after VEGFA treatment, positively correlated with BCNS keratinocytes after rapamycin treatment (data not shown).

### Biological differences attributed to rapamycin effects in BCNS and normal fibroblasts

The GSEA pointed to genes upregulated by activation of PI3K/AKT/mTOR pathway and mTORC1 complex, as well as genes involved in mitotic spindle assembly, DNA repair, G2/M checkpoint, and genes regulated by MYC which appear to be in agreement with the transcriptomic profiles of BCNS fibroblasts post rapamycin treatment (Figure 3B, Supplementary Files 3–4). Additionally, genes upregulated by APC knockout, RNAi mediated ELK3 knockdown in response to hypoxia, MYC and IRF4 targets, along with genes down regulated in lesional skin biopsies from mycosis fungoides patients compared to normal skin samples positively correlated with the results on treated BCNS fibroblasts. Oncogenic signatures comprising of up-regulated genes in primary keratinocytes from RB1 and RBL2 skin specific knockout, genes upregulated upon EED or EZH2 knockdown, MYC overexpression, stimulation with Shh, and genes upregulated by everolimus are found to be correlated with treated BCNS fibroblasts. Also, significant immunologic signatures were enriched in the treated BCNS fibroblasts.

Furthermore, GSEA indicated genes involved in glycogenesis and gluconeogenesis and genes upregulated by PI3K/AKT/mTOR pathway and mTORC1 complex activation, genes important for and involved in mitotic spindle assembly, DNA repair, G2/M checkpoint, genes responding to estrogen and genes regulated by MYC [49] were negatively correlated with the transcriptomic profiles of BCNS fibroblasts post rapamycin treatment. Additionally, genes expressed in multipotent progenitors, genes upregulated by APC or BCL3 knockout or RNAi mediated ELK3 knockdown in response to hypoxia, MYC and IRF4 targets, along with genes downregulated in lesional skin biopsies from mycosis fungoides patients compared to normal skin samples and genes upregulated in peripheral blood monocytes of Sezary syndrome, were negatively correlated with rapamycin-treated BCNS fibroblasts. Also, oncogenic signatures comprising of YAP [50, 51] conserved signature, up-regulated genes in RB1 and RBL2 skin specific knockout primary keratinocytes, genes upregulated upon EED or EZH2 knockdown, MYC overexpression, stimulation with Shh, and genes upregulated by everolimus, were found to be negatively correlated with treated BCNS fibroblasts. Gene sets consisting of downregulated genes upon SRC overexpression, RPS14 knockdown, HOXA9 knockdown, and VEGFA treatment were also negatively correlated with rapamycin treated BCNS fibroblasts. On other hand, significant immunologic signatures were enriched in untreated BCNS fibroblasts.

### Ingenuity Pathway Analysis (IPA) shows distinct signaling pathways enriched in BCNS samples treated with rapamycin

**Figure 4:**
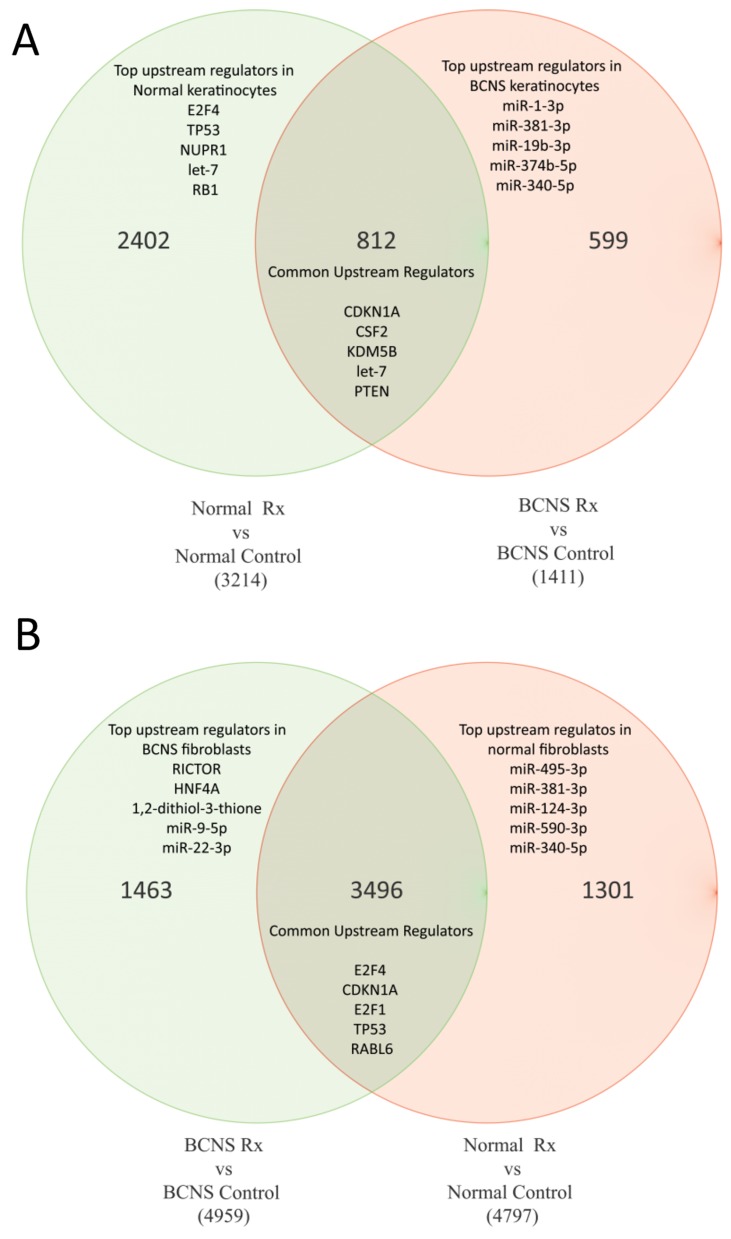
Venn diagrams of genes commonly altered in both normal and BCNS group, with or without rapamycin treatment Normal treated (Rx) samples were compared to normal control samples, and these samples were then compared BCNS treated (Rx) samples to BCNS control samples. **(A)** Keratinocyte samples. **(B)** Fibroblast samples.

To study whether rapamycin treatment affected cells derived from BCNS patients with *PTCH1* mutation differently than normal samples, we generated a gene list of the top differentially regulated and statistically significant genes (cut-off p-value with FDR <0.05, FC>1.3 or <-1.3), including the top genes as listed in Tables 3 and 4 (the entire gene list is available in Supplementary File 4). Thus, a total of 4797 genes were differentially expressed in normal fibroblasts and expression of 3214 genes were significantly altered in normal keratinocytes after rapamycin treatment. In the BCNS group, a total of 4959 genes and 1411 genes were differentially expressed in fibroblasts and keratinocytes, respectively after treatment with rapamycin. To tease out genes that were usually altered in both normal and BCNS groups following rapamycin treatment, we used Venn diagrams (Figure 4) to generate gene lists for further analysis.

**Table 3:** List of top genes differentially regulated after rapamycin treatment in keratinocytes and fibroblasts derived from normal individuals

Gene Symbol	Gene title (HGNC approved)	Fold change	p-value
***Genes up-regulated in normal keratinocytes after rapamycin treatment***			
KLHL24	kelch-like family member 24	11.2986	0.000701
C5orf41	CREB3 regulatory factor	7.59837	0.000914
IRF6	interferon regulatory factor 6	7.09155	0.003863
ATF3	activating transcription factor 3	6.42457	6.31E-05
GPNMB	glycoprotein (transmembrane) nmb	6.19196	0.000485
NEAT1	nuclear paraspeckle assembly transcript 1 (non-protein coding)	5.539	0.001790
MALAT1	metastasis associated lung adenocarcinoma transcript 1 (non-protein coding)	5.28172	0.000797
IRS2	insulin receptor substrate 2	4.58514	0.000667
DUSP10	dual specificity phosphatase 10	4.47873	0.000829
S100P	S100 calcium binding protein P	4.22571	3.44E-09
***Genes down-regulated in normal keratinocytes after rapamycin treatment***			
RRM2	ribonucleotide reductase M2	-13.9567	0.001023
DTL	denticleless E3 ubiquitin protein ligase homolog (Drosophila)	-12.2969	0.003649
CCNE2	cyclin E2	-10.7273	2.43E-05
UHRF1	ubiquitin-like with PHD and ring finger domains 1	-10.2451	0.000128
MCM10	minichromosome maintenance complex component 10	-9.9609	0.002114
CDC6	cell division cycle 6	-9.89019	0.002154
THBS1	thrombospondin 1	-8.83678	6.24E-05
SHCBP1	SHC SH2-domain binding protein 1	-7.3742	4.93E-06
DHFR	dihydrofolate reductase	-7.32771	0.004707
ZWINT	ZW10 interactor	-7.20764	0.001275
***Genes up-regulated in normal fibroblasts after rapamycin treatment***			
ADH1B	alcohol dehydrogenase 1B (class I), beta polypeptide	17.4006	7.18E-06
ITGB8	integrin, beta 8	6.98181	0.000417
DIO2	deiodinase, iodothyronine, type II	6.75995	5.31E-05
FGF7	fibroblast growth factor 7	5.35228	0.001493
EFEMP1	EGF containing fibulin-like extracellular matrix protein 1	5.18771	0.000186
GRIA1	glutamate receptor, ionotropic, AMPA 1	5.17074	9.47E-09
SLC40A1	solute carrier family 40 (iron-regulated transporter), member 1	5.06774	0.000937
C13orf15	regulator of cell cycle	4.82343	7.65E-05
FMO2	flavin containing monooxygenase 4 (non-functional)	4.74587	2.43E-07
SVEP1	sushi, von Willebrand factor type A, EGF and pentraxin domain containing 1	4.70169	1.93E-06
***Genes down-regulated in normal fibroblasts after rapamycin treatment***			
ANLN	anillin, actin binding protein	-40.9022	6.14E-06
PBK	PDZ binding kinase	-38.6028	2.70E-06
DLGAP5	discs, large (Drosophila) homolog-associated protein 5	-35.7893	7.76E-08
RRM2	ribonucleotide reductase M2	-34.5474	2.81E-06
HMMR	hyaluronan-mediated motility receptor (RHAMM)	-30.7856	1.68E-07
TOP2A	topoisomerase (DNA) II alpha 170kDa	-30.641	5.36E-07
SHCBP1	SHC SH2-domain binding protein 1	-30.4333	7.90E-09
CEP55	centrosomal protein 55kDa	-29.9081	1.91E-09
TTK	TTK protein kinase	-29.053	1.60E-08
NDC80	NDC80 kinetochore complex component	-28.6074	5.35E-08

**Table 4:** List of top genes differentially regulated after rapamycin treatment in keratinocytes and fibroblasts derived from BCNS individuals

Gene Symbol	Gene title (HGNC approved)	Fold change	p-value
***Genes up-regulated in BCNS keratinocytes after rapamycin treatment***			
KLHL24	kelch-like family member 24	7.63805	0.000303
GPNMB	glycoprotein (transmembrane) nmb	5.38456	0.000811
ATF3	activating transcription factor 3	4.94199	0.000227
GABARAPL1	GABA(A) receptor-associated protein like 1	3.95871	0.000281
IRS2	insulin receptor substrate 2	3.88798	0.001592
JMY	junction mediating and regulatory protein, p53 cofactor	3.36795	1.90E-06
ADCK3	aarF domain containing kinase 3	3.21844	9.14E-05
BIRC3	baculoviral IAP repeat containing 3	3.11232	0.000889
ZBTB10	zinc finger and BTB domain containing 10	3.02502	0.002266
NCRNA00219	EPB41L4A antisense RNA 1	2.94874	4.48E-07
***Genes down-regulated in BCNS keratinocytes after rapamycin treatment***			
THBS1	thrombospondin 1	-5.00466	0.001087
GJB2	gap junction protein, beta 2, 26kDa	-3.99488	0.001130
NAV3	neuron navigator 3	-3.86258	0.000239
ABAT	4-aminobutyrate aminotransferase	-3.1763	1.41E-06
SHCBP1	SHC SH2-domain binding protein 1	-3.16137	0.001208
CDC25A	cell division cycle 25A	-3.08918	0.001806
CXCL14	chemokine (C-X-C motif) ligand 14	-2.93124	5.30E-06
SORL1	sortilin-related receptor, L(DLR class) A repeats containing	-2.81784	0.001444
KRT1	keratin 1, type II	-2.77983	0.001714
CDKN3	cyclin-dependent kinase inhibitor 3	-2.75698	0.000109
***Genes up-regulated in BCNS fibroblasts after rapamycin treatment***			
ADH1B	alcohol dehydrogenase 1B (class I), beta polypeptide	14.5357	9.23E-06
TXNIP	thioredoxin interacting protein	6.7023	0.004666
ITGB8	integrin, beta 8	5.53155	0.001337
GLDN	gliomedin	5.28474	0.000403
DIO2	deiodinase, iodothyronine, type II	5.08305	0.000173
GRIA1	glutamate receptor, ionotropic, AMPA 1	4.61537	1.38E-08
FGF9	fibroblast growth factor 9	4.57488	0.002351
C13orf15	regulator of cell cycle	4.53467	7.38E-05
SVEP1	sushi, von Willebrand factor type A, EGF and pentraxin domain containing 1	4.33477	2.99E-06
SLC40A1	solute carrier family 40 (iron-regulated transporter), member 1	4.24931	0.002374
***Genes down-regulated in BCNS fibroblasts after rapamycin treatment***			
ANLN	anillin, actin binding protein	-48.0347	4.14E-06
PBK	PDZ binding kinase	-45.1184	1.81E-06
KIF20A	kinesin family member 20A	-37.2298	2.83E-06
DLGAP5	discs, large (Drosophila) homolog-associated protein 5	-36.5714	7.41E-08
CCNB1	cyclin B1	-35.2177	3.43E-06
RRM2	ribonucleotide reductase M2	-29.9324	4.66E-06
NDC80	NDC80 kinetochore complex component	-25.8672	8.81E-08
CEP55	centrosomal protein 55kDa	-25.5206	2.31E-09
BIRC5	baculoviral IAP repeat containing 5	-25.4299	1.16E-07
HMMR	hyaluronan-mediated motility receptor (RHAMM)	-25.2382	4.16E-07

### IPA core analysis indicates effect of rapamycin treatment on keratinocyte and fibroblasts

**Figure 5:**
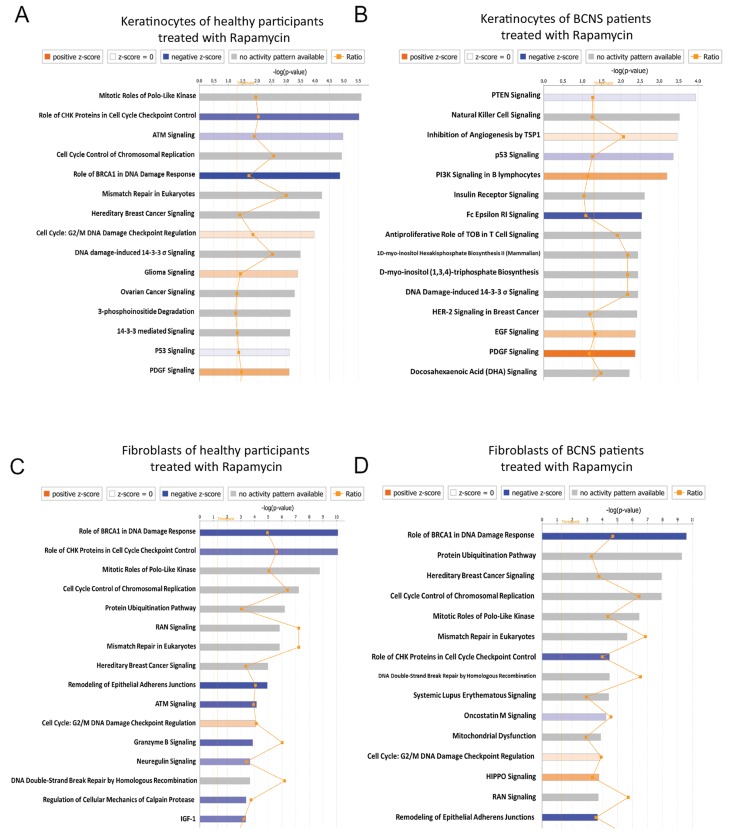
IPA core analysis indicates effect of rapamycin treatment on keratinocyte and fibroblasts **(A)** Keratinocytes of healthy participants treated with rapamycin. **(B)** Keratinocytes of BCNS patients treated with rapamycin. **(C)** Fibroblasts of healthy participants treated with rapamycin. **(D)** Fibroblasts of BCNS patients treated with rapamycin.

Here we explored the genes/signaling pathways differentially expressed in response to rapamycin. To this end, we performed core IPA analysis on comprehensive gene lists for fibroblasts and keratinocyte, including prioritized gene lists that were generated via the Venn diagram in Figure 4. The IPA core analysis showed enrichment of pathways that comprised mitotic role of Polo-like Kinases, role of CHK proteins in cell cycle checkpoint control, ATM signaling, cell cycle control of chromosomal replication and role of BRCA1 in DNA damage response as top canonical pathways, based on focus genes present among the 3214 genes that are differentially regulated in normal keratinocytes in response to rapamycin (Figure 5A, Table 5A). A Similar IPA core analysis of BCNS keratinocytes treated with rapamycin resulted in enrichment of PTEN signaling, Natural killer cell signaling, inhibition of angiogenesis by TSP1, p53 signaling and PI3K signaling in B lymphocytes as top canonical pathways, based on focus genes present in 1411 differentially regulated genes after rapamycin treatment (Figure 5B, Table 5B).

**Table 5A:** Keratinocytes of healthy participants treated with rapamycin

Ingenuity Canonical Pathways	-log(p-value)	Molecules
Mitotic Roles of Polo-Like Kinase	5.6	PPP2R5E, KIF23, CHEK2, PPP2R4, CDC25A, PRC1, CDC20, PPP2R3A, CCNB2, KIF11, SMC3, PPP2R2A, WEE1, ANAPC11, PKMYT1, PLK4, ANAPC7, FZR1, CDC23, PLK1, CDK1, CCNB1, FBXO5
Role of CHK Proteins in Cell Cycle Checkpoint Control	5.53	SLC19A1, PPP2R5E, E2F5, BRCA1, RFC3, CHEK2, PPP2R4, CDC25A, MDC1, PPP2R3A, PPP2R2A, RAD1, RAD9A, RFC5, CDKN1A, RFC2, RFC4, ATMIN, PLK1, CDK1, CHEK1
ATM Signaling	4.97	JUN, BRCA1, CBX5, CREB1, CHEK2, CDC25A, GADD45B, MDC1, CCNB2, TDP1, SMC2, SMC3, FANCD2, RAD9A, CDKN1A, MAPK8, RAD51, GADD45G, CDK1, CCNB1, CHEK1
Cell Cycle Control of Chromosomal Replication	4.92	CDK7, CHEK2, MCM7, ORC6, ORC1, CDC45, RPA3, MCM3, MCM6, CDK6, MCM4, MCM5, CDC6
Role of BRCA1 in DNA Damage Response	4.87	RFC3, CHEK2, SMARCA4, MDC1, MSH6, RFC2, CDKN1A, FANCG, RBL1, RFC4, SLC19A1, E2F5, BRCA1, ATF1, SMARCE1, FANCM, FANCD2, BRD7, RFC5, ARID2, BRCA2, RAD51, PLK1, MSH2, CHEK1
Mismatch Repair in Eukaryotes	4.24	SLC19A1, FEN1, RFC3, EXO1, MSH6, RFC5, RFC2, RFC4, MSH2
Hereditary Breast Cancer Signaling	4.16	RFC3, AKT3, CHEK2, PIK3C2A, TUBG1, GADD45B, SMARCA4, PIK3CA, MSH6, RFC2, CDKN1A, FANCG, CDK6, RFC4, GADD45G, SLC19A1, BRCA1, PIK3R4, SMARCE1, FANCM, AKT2, WEE1, POLR2H, FANCD2, BRD7, RFC5, ARID2, BRCA2, RAD51, CDK1, CCNB1, MSH2, CHEK1
Cell Cycle: G2/M DNA Damage Checkpoint Regulation	3.98	TOP2A, BRCA1, YWHAQ, CDK7, CHEK2, AURKA, PTPMT1, CCNB2, WEE1, PKMYT1, SKP2, CDKN1A, YWHAB, PLK1, CDK1, CCNB1, CHEK1
DNA damage-induced 14-3-3σ Signaling	3.49	CCNE2, BRCA1, AKT3, RAD9A, CCNB2, AKT2, RAD1, CDK1, CCNB1
Glioma Signaling	3.41	SOS2, AKT3, PIK3C2A, CALM1 (includes others), CAMK2D, PDGFB, PIK3CA, CDKN2D, CDKN1A, GRB2, SIN3A, PA2G4, RBL1, CDK6, E2F5, EGFR, PRKD3, PIK3R4, CAMK1D, AKT2, IGF1R, PRKCA, MAPK1, TFDP1, PRKCG
Ovarian Cancer Signaling	3.29	EDNRA, AKT3, PIK3C2A, WNT5A, FZD5, WNT11, PIK3CA, FZD1, MSH6, EDN1, SIN3A, PA2G4, WNT6, VEGFC, PRKAR2A, BRCA1, EGFR, TCF3, CGA, TCF4, PIK3R4, AKT2, CD44, BRAF, PTGS2, MAPK1, BRCA2, RAD51, TFDP1, MSH2, GJA1
3-phosphoinositide Degradation	3.15	DUSP10, PPP2R5E, NUDT1, MTMR4, NUDT3, PPM1F, PPP2R4, PPP2R3A, MTMR3, STYXL1, OCRL, INPP5D, DUSP23, DUSP1, PPIP5K1, SYNJ2, MTMR6, PXYLP1, INPP5F, PTPMT1, PTPN2, CDC25A, PTPRO, PAWR, DUSP2, ACP1, TMEM55B, NUDT4, HACD2, PPP1R13B, NUDT15, IGBP1
14-3-3-mediated Signaling	3.13	JUN, YWHAQ, AKT3, PIK3C2A, TUBB3, TUBG1, YAP1, TUBA1C, VIM, TUBA1A, SNCA, PIK3CA, GRB2, MAPK8, MAPT, YWHAB, FOXO1, TUBA3C/TUBA3D, PRKD3, PIK3R4, AKT2, TUBB4B, PRKCA, TUBA1B, MAPK1, TUBA4A, BAX, PRKCG
p53 Signaling	3.12	JUN, AKT3, TP53AIP1, CHEK2, PIK3C2A, GADD45B, THBS1, PIK3CA, CDKN1A, PLAGL1, MAPK8, GADD45G, BRCA1, TP63, PIK3R4, JMY, BIRC5, ST13, AKT2, CASP6, HIF1A, PPP1R13B, BAX, ADCK3, CHEK1
PDGF Signaling	3.11	JUN, EIF2AK2, SOS2, PIK3C2A, INPP5F, CRKL, PIK3R4, CSNK2A2, JAK1, PDGFB, CSNK2A1, ABL2, OCRL, INPP5D, PIK3CA, ACP1, PRKCA, MAPK1, GRB2, SYNJ2, MAPK8

**Table 5B:** Keratinocytes of BCNS patients treated with rapamycin

Ingenuity Canonical Pathways	-log(p-value)	Molecules
PTEN Signaling	3.94	FGFR2, AKT3, INPP5F, BCL2L11, TGFBR3, CSNK2A2, AKT2, CSNK2A1, BMPR1B, H2BFM, OCRL, INPP5D, PIK3CA, CDKN1A, MAPK1, SYNJ2, ITGA4, TGFBR1
Natural Killer Cell Signaling	3.52	PIK3R6, AKT3, INPP5F, LILRB1, AKT2, VAV2, FYN, OCRL, INPP5D, PIK3CA, PRKCA, TYROBP, SH3BP2, MAPK1, SYNJ2, NCK1
Inhibition of Angiogenesis by TSP1	3.46	JUN, FYN, AKT3, MMP9, MAPK1, TGFBR1, THBS1, AKT2
p53 Signaling	3.36	JUN, PIK3R6, AKT3, TP53AIP1, JMY, BIRC5, ST13, THBS1, AKT2, PIK3CA, CDKN1A, PLAGL1, BAX, ADCK3, CHEK1
PI3K Signaling in B Lymphocytes	3.2	JUN, AKT3, IRS2, CD79B, CHP1, ATF6, AKT2, PPP3CC, VAV2, ATF3, MALT1, FYN, INPP5D, PIK3CA, PLEKHA3, MAPK1, SH2B2
Insulin Receptor Signaling	2.62	PIK3R6, AKT3, IRS2, INPP5F, SCNN1A, AKT2, H2BFM, FYN, OCRL, INPP5D, PIK3CA, GYS1, MAPK1, SYNJ2, SH2B2, NCK1
Fc Epsilon RI Signaling	2.55	PIK3R6, AKT3, INPP5F, AKT2, VAV2, FYN, OCRL, INPP5D, PIK3CA, PRKCA, MAPK1, SYNJ2, PLA2G12A, PLA2G4A
Antiproliferative Role of TOB in T Cell Signaling	2.53	SMAD2, SKP2, MAPK1, CCNA2, TGFBR1, SMAD4
1D-myo-inositol Hexakisphosphate Biosynthesis II (Mammalian)	2.44	OCRL, INPP5D, INPP5F, ITPKB, SYNJ2
D-myo-inositol (1,3,4)-trisphosphate Biosynthesis	2.44	OCRL, INPP5D, INPP5F, ITPKB, SYNJ2
DNA damage-induced 14-3-3σ Signaling	2.44	AKT3, CCNB2, AKT2, RAD1, CCNB1
HER-2 Signaling in Breast Cancer	2.42	H2BFM, PIK3R6, AKT3, ITGB5, PIK3CA, ITGB6, PARD3, PRKCA, PARD6G, CDKN1A, AKT2
EGF Signaling	2.37	JUN, PIK3R6, AKT3, PIK3CA, PRKCA, CSNK2A2, MAPK1, AKT2, CSNK2A1
PDGF Signaling	2.37	JUN, PIK3R6, OCRL, INPP5D, PIK3CA, INPP5F, PRKCA, CSNK2A2, MAPK1, SYNJ2, CSNK2A1
Docosahexaenoic Acid (DHA) Signaling	2.22	H2BFM, PIK3R6, AKT3, PIK3CA, APP, BAX, AKT2

In the case of the normal fibroblasts, an IPA core analysis of 4797 differentially regulated genes in response to rapamycin resulted in enrichment of role of BRCA1 in DNA damage response, role of CHK proteins in cell cycle checkpoint control, mitotic roles of Polo-like Kinases, cell cycle control of chromosomal replication and protein ubiquitination pathway as canonical signaling pathways (Figure 5C, Table 5C). On the other hand, a core analysis of 4959 genes enriched in rapamycin treated BCNS fibroblasts pointed to top canonical pathways that included those playing a role of BRCA1 in DNA damage response, protein ubiquitination pathway, hereditary breast cancer signaling, cell cycle control of chromosomal replication and mitotic roles of Polo-like Kinases (Figure 5D, Table 5D). Although it may appear that there was some overlap among canonical signaling pathways between normal and BCNS samples, the focus molecules in these pathways were different which would affect the direction of the pathway. BCNS keratinocytes had the most distinct canonical pathway signaling. The p-values and molecules for top canonical pathways are shown (Table 5A–5D); a comprehensive list is shown in Supplementary File 5.

**Table 5C:** Fibroblasts of healthy participants treated with rapamycin

Ingenuity Canonical Pathways	-log(p-value)	Molecules
Role of BRCA1 in DNA Damage Response	10.1	RFC3, FANCB, PBRM1, ATRIP, E2F1, BARD1, SMARCA4, MDC1, E2F3, NBN, TOPBP1, STAT1, MSH6, RFC2, FANCG, RPA1, RBL1, RFC4, MRE11A, SLC19A1, BRCA1, BRE, BRCC3, HLTF, FANCM, SMARCC2, RBBP8, BLM, BRIP1, RFC1, FANCD2, RFC5, FANCA, GADD45A, BRCA2, RAD51, ARID1A, PLK1, MSH2, CHEK1
Role of CHK Proteins in Cell Cycle Checkpoint Control	10.1	RFC3, E2F1, PPP2R3B, CLSPN, MDC1, PPP2R3A, E2F3, PCNA, PPP2R5C, CDC25C, NBN, RAD9A, RFC2, RPA1, PPP2CB, RFC4, MRE11A, PPP2CA, SLC19A1, BRCA1, CDC25A, CDK2, PPP2R2A, RAD1, HUS1, RFC1, RFC5, PPP2R5D, RAD17, PLK1, CDK1, CHEK1
Mitotic Roles of Polo-Like Kinase	8.76	KIF23, SMC1A, ESPL1, PPP2R3B, PPP2R3A, CCNB2, KIF11, ANAPC1, PPP2R5C, CDC25C, ANAPC5, PKMYT1, PPP2CB, HSP90AA1, PPP2CA, CDC27, CDC25A, PRC1, CDC20, PPP2R2A, CDC7, RAD21, PPP2R5D, PLK4, PLK2, HSP90AB1, PTTG1, FZR1, PLK1, CDK1, CCNB1, FBXO5, CDC25B
Cell Cycle Control of Chromosomal Replication	7.22	MCM7, MCM2, CDK2, ORC6, DBF4, CDC7, ORC1, CDC45, RPA1, RPA3, MCM3, MCM6, CDT1, CDK6, MCM4, MCM5, CDC6, ORC3
Protein Ubiquitination Pathway	6.2	HSPA2, UBE2M, DNAJC5, PSMD6, SMURF2, USP7, ANAPC1, HSPB2, ANAPC5, USP1, USP46, PSMA7, DNAJC9, UBE2V2, UCHL5, PSMB2, PSMA1, TRAF6, TCEB2, UBE2L3, UBE2H, USP14, UBE2G2, PSMA6, UBE2V1, PSME1, PSMD12, DNAJC18, UBE2J2, PSMC5, SUGT1, PSMD1, USP13, DNAJB11, UBE2C, HLA-C, HLA-B, NEDD4L, USP47, USP19, DNAJA1, DNAJB13, DNAJC11, THOP1, HSPD1, DNAJC4, USP5, PSME2, CBL, PSMD11, UBE2S, PSMD9, TCEB1, PSMD14, PSMC1, USP24, PSMD2, HSP90AA1, UBE2R2, BRCA1, HSPB11, UCHL1, PSMC3, UBR1, UBE2N, CDC20, USP10, HSPA4, NEDD4, UBE2B,HLA-A, UBE2L6, UBE2I, UCHL3, PSMD13, DNAJB4, HSP90AB1, FZR1, USP53
RAN Signaling	5.82	KPNA1, CSE1L, RANGAP1, KPNA2, XPO1, RCC1, KPNA3, IPO5, KPNA6, RAN, RANBP1, KPNB1
Mismatch Repair in Eukaryotes	5.82	SLC19A1, RFC1, FEN1, RFC3, EXO1, MSH6, RFC5, RFC2, RPA1, RFC4, PCNA, MSH2
Hereditary Breast Cancer Signaling	4.97	RFC3, FANCB, PBRM1, HDAC8, TUBG1, E2F1, BARD1, SMARCA4, PIK3R1, H2AFX, CDC25C, NBN, MSH6, RFC2, FANCG, RPA1, CDK6, RFC4, NPM1, MRE11A, SLC19A1, BRCA1, HDAC2, PIK3R3, POLR2E, HLTF, FANCM, SMARCC2, BLM, RFC1, FANCD2, POLR2D, RFC5, CCND1, FANCA, GADD45A, BRCA2, RAD51, HRAS, ARID1A, CDK1, CCNB1, MSH2, CHEK1
Remodeling of Epithelial Adherens Junctions	4.94	IQGAP1, ACTR2, VCL, TUBB3, TUBB6, MAPRE1, TUBG1, HGS, TUBA1C, ARPC5L, APC, DNM1L, TUBA1A, MET, TUBA3C/TUBA3D, ARPC1A, CTNNA1, TUBB, ACTN1, NME1, TUBB4B, ARPC2, DNM2, RALA, TUBA1B, TUBA4A, ARF6
ATM Signaling	4.15	JUN, BRCA1, CBX5, SMC1A, CDK2, CDC25A, MDC1, CCNB2, TDP1, SMC2, H2AFX, CDC25C, NBN, BLM, TRIM28, FANCD2, RAD9A, MAPK8, GADD45A, RAD51, MRE11A, CDK1, CCNB1, CHEK1
Cell Cycle: G2/M DNA Damage Checkpoint Regulation	4.1	TOP2A, BRCA1, YWHAQ, YWHAG, AURKA, CKS2, CCNB2, YWHAH, CDC25C, BORA, PKMYT1, GADD45A, CKS1B, HIPK2, PRKDC, YWHAZ, PLK1, CDK1, CCNB1, CHEK1, CDC25B
Granzyme B Signaling	3.87	PARP1, APAF1, LMNB1, ENDOG, DFFA, PRKDC, CYCS, LMNB2, NUMA1, CASP8
Neuregulin Signaling	3.68	SOS2, ERBB2IP, ITGA2, PIK3R1, PTPN11, STAT5A, GRB2, DCN, CRK, HSP90AA1, PSEN1, RPS6KB2, TGFA, HBEGF, EGFR, PIK3R3, NRG1, RNF41, SOS1, PLCG2, PDPK1, DLG4, PRKCA, MAPK1, ITGA4, PLCG1, HSP90AB1, STAT5B, HRAS, ITGA5
DNA dsBreak Repair by Homologous Recombination	3.66	BRCA1, POLA1, GEN1, RPA1, LIG1, BRCA2, RAD51, MRE11A, NBN
Regulation of Cellular Mechanics by Calpain Protease	3.39	EGFR, TLN1, VCL, ITGA2, EZR, CDK2, PXN, ACTN1, TLN2, PTK2, CAPN5, CCND1, MAPK1, GRB2, ITGA4, CCNA2, CDK6, HRAS, ITGA5, CDK1, CAPN2

**Table 5D:** Fibroblasts of BCNS patients treated with rapamycin

Ingenuity Canonical Pathways	-log(p-value)	Molecules
Role of BRCA1 in DNA Damage Response	9.59	POU2F1, RFC3, FANCB, PBRM1, ATRIP, BARD1, SMARCA4, SMARCB1, NBN, DPF1, STAT1, MSH6, RFC2, FANCG, RPA1, RBL1, RFC4, MRE11A, SLC19A1, BRCA1, SMARCD1, BRE, MLH1, BABAM1, BRCC3, HLTF, SMARCE1, FANCM, SMARCC2, RBBP8, BRIP1, FANCD2, RFC5, FANCA, GADD45A, BRCA2, RAD51, PLK1, MSH2, CHEK1
Protein Ubiquitination Pathway	9.27	HSPA2, HSPA1L, UBE2M, PSMD3, DNAJC5, PSMD6, SMURF2, PSMB5, ANAPC1, PSMC4, ANAPC5, USP1, USP46, PSMA7, DNAJC9, PSMB7, UBE2V2, UCHL5, PSMA2, PSMB2, UBE2A, PSMA1, USP32, TRAF6, UBE2L3, PSMA4, UBE2H, USP14, UBE2G2, PSMA6, HSPB8, UBE2V1, PSME1, DNAJB5, PSMC2, PSMD12, DNAJC18, UBE4A, UBE2J2, PSMC5, SUGT1, PSMD1, USP13, UBE2C, HLA-C, HLA-B, RBX1, USP47, DNAJA1, DNAJB13, PSMD7, MED20, THOP1, HSPD1, DNAJB12, DNAJC4, USP5, PSME2, CBL, PSMD11, UBE2S, PSMD4, PSMD9, TCEB1, PSMD14, PSMC1, PSMB3, PSMD2, PSMD5, UBE2R2, PSMA5, BRCA1, HSPB11, PSMC3, UBR1, UBE2N, CDC20, USP10, HSPA4, USP39, NEDD4, HLA-A, UBE2D2, UCHL3, PSMD13, DNAJB4, PSMD8, PAN2, FZR1, USP53
Hereditary Breast Cancer Signaling	7.93	RFC3, HDAC8, TUBG1, POLR2F, BARD1, SMARCA4, POLR2K, SMARCB1, NBN, DPF1, RPA1, RFC4, MRE11A, HDAC1, SLC19A1, PIK3R3, SMARCD1, POLR2E, MLH1, HLTF, SMARCE1, SMARCC2, NRAS, FANCD2, POLR2D, CCND1, FANCA, GADD45A, BRCA2, MSH2, FANCB, PBRM1, PIK3R1, H2AFX, CDC25C, MSH6, RFC2, FANCG, CDK6, NPM1, BRCA1, HDAC2, SFN, RRAS2, FANCM, POLR2H, RFC5, RAD51, HRAS, CCNB1, CDK1, CHEK1
Cell Cycle Control of Chromosomal Replication	7.92	MCM7, MCM2, CDK2, ORC6, CDK5, DBF4, CDC7, ORC1, CDC45, RPA1, RPA3, MCM3, MCM6, CDT1, CDK6, MCM4, MCM5, CDC6, ORC3
Mitotic Roles of Polo-Like Kinase	6.44	KIF23, TGFB1, SMC1A, ESPL1, PPP2R4, PPP2R3A, CCNB2, KIF11, ANAPC1, PPP2R5C, CDC25C, ANAPC5, PKMYT1, ANAPC7, PPP2CA, CDC27, CDC25A, PRC1, CDC20, CDC7, RAD21, PPP2R5D, PLK4, PLK2, PTTG1, FZR1, PLK1, CDK1, CCNB1, FBXO5
Mismatch Repair in Eukaryotes	5.64	SLC19A1, FEN1, RFC3, EXO1, MSH6, RFC5, RFC2, RPA1, MLH1, RFC4, PCNA, MSH2
Role of CHK Proteins in Cell Cycle Checkpoint Control	4.48	SLC19A1, BRCA1, RFC3, PPP2R4, CDK2, CDC25A, CLSPN, PPP2R3A, PCNA, CDC25C, HUS1, PPP2R5C, NBN, RAD9A, RFC5, RFC2, PPP2R5D, RPA1, RFC4, MRE11A, PLK1, CDK1, PPP2CA, CHEK1
DNA dsBreak Repair by Homologous Recombination	4.47	BRCA1, POLA1, RAD52, GEN1, RPA1, LIG1, BRCA2, RAD51, MRE11A, NBN
Systemic Lupus Erythematosus Signaling	4.42	SNRPB2, LSM2, SNRNP40, SNRPD1, HNRNPC, FCER1G, LSM12, PRPF19, SNRNP25, SNRPA, PIK3R3, LSM8, HLAG, PRPF3, LSM1, NRAS, PRPF38A, HLA-C, HLA-B, PRPF31, JUN, PIK3R1, CBL, IL37, SNRNP200, LSM5, SNRPA1, PRPF8, SNRNP27, GRB2, IGHG1, LSM14B, SNRPG, NFATC4, HLA-E, RRAS2, SNRPE, SNRPC, PRPF4, LSM4, SOS1, SART1, SNRPN, HLA-A, NHP2L1, SNRPF, TXNL4A, MAPK1, PRPF40A, EFTUD2, SNRPD3, HRAS, LSM3, ZCRB1, HNRNPA2B1, PPIH, SNRPB
Oncostatin M Signaling	4.24	MT2A, RRAS2, PLAU, SOS1, OSMR, NRAS, MMP3, STAT3, STAT1, STAT5A, TIMP3, MAPK1, GRB2, EPAS1, STAT5B, HRAS, MMP1
Mitochondrial Dysfunction	3.89	VDAC3, ATP5F1, COX5A, MAPK10, MTND2, SDHB, NDUFA8, NDUFAB1, NDUFA9, SDHD, ATP5C1, BACE1, ATP5B, SDHC, UQCRC1, HTRA2, APP, NDUFB8, CPT1B, CYCS, VPS9D1, CYC1, SOD2, PRDX5, NDUFS6, AIFM1, MAOB, ATP5G3, COX11, VDAC1, COX7A1, CPT1A, GLRX2, NDUFV3, PRDX3, GPX4, NDUFA6, NDUFV2, MAOA, PSEN1, HSD17B10, ATP5G1, SURF1, PDHA1, NDUFS1, NDUFB6, CYB5A, NDUFA3, ATP5J2, COX7C
Cell Cycle: G2/M DNA Damage Checkpoint Regulation	3.86	TOP2A, BRCA1, YWHAQ, SFN, YWHAG, AURKA, YWHAE, CKS2, CCNB2, YWHAH, CDC25C, BORA, PKMYT1, GADD45A, CKS1B, PRKDC, YWHAZ, PLK1, CDK1, CCNB1, CHEK1
HIPPO signaling	3.78	PPP1CC, YWHAQ, PARD3, PPP2R4, PPP2R3A, SMAD4, YWHAE, YWHAH, PPP2R5C, LLGL1, CSNK1D, PPP1R14B, ITCH, MOB1A, PPP2CA, CSNK1E, SFN, NF2, YWHAG, PPP1R12A, PPP1CA, TJP2, WWTR1, CD44, DLG4, RASSF1, PPP1CB, PPP2R5D, SAV1, YWHAZ, STK4
RAN Signaling	3.73	KPNA1, CSE1L, RANGAP1, KPNA2, KPNA3, IPO5, KPNA6, RAN, RANBP1, KPNB1

In order to understand how canonical pathways that are uniquely enriched in normal or BCNS specimens respond differently to rapamycin due to differentially regulated genes, we dissected the gene list guided by the Venn diagram (Figure 4) and performed core analysis on each group. The canonical pathways enriched in both normal and BCNS keratinocytes, based on 812 common genes, were p53 signaling, DNA damage induced 14-3-3 signaling, EGF signaling, IL-3 signaling and PTEN signaling. The IPA core analysis of the 812 differentially regulated genes common to both BCNS and normal keratinocytes after rapamycin treatment predicted CDKN1A, KDM5B, let-7 and PTEN to be activated upstream regulators while CSF2 to be inhibited. The regulator networks associated with IDs correspond to diseases and function namely cell death of squamous cell carcinoma cell lines. The top up-regulated molecules were ATF3, GPNMB, KLHL24, IRS2, S100P, OTUD1, GABARAPL3, HIST3H2A, EPB41L4A-AS1, LIMA1, TIAM2, JMY and ADCK3. The top down-regulated molecules are THBS1, SHCBP1, CDC25A, NAV3, ZNF367, ABAT, NCAPG, PLAGL1, TTK, GJB2, CXCL14, SORL1, CDKN3, RAB7B.

The canonical pathways enriched in normal keratinocytes only, based on 2402 genes analyzed, were mitotic roles of polo-like kinases, mismatch repair in eukaryotes, role of CHK proteins in cell cycle checkpoint control, role of BRCA1 in DNA damage response and cell cycle control of chromosomal replication. The top up-regulated molecules only in normal keratinocytes are KLHL24, CREBRF, IRF6, NEAT1, MALAT1, DUSP10, TXNIP, DDX6, CITED2, ZMAT3. The top down-regulated molecules are RRM2, DTL, CCNE2, UHRF1, MCM10, CDC6, DHFR, ZWINT, GINS2, PTGS2 in normal keratinocytes in response to rapamycin. It is of interest that although the upstream regulator E2F4 has no activation prediction, TP53, NUPR1, let-7 and RB1 are predicted to be active upstream regulators in normal keratinocytes.

Hepatic fibrosis/hepatic stellate cell activation, HIPPO signaling, natural killer cell signaling, galactose degradation (Leloir Pathway) and ILK signaling were identified as the top canonical pathways unique to the 599 focus genes in the BCNS keratinocytes samples after rapamycin treatment. The top up-regulated molecules were BIRC3, ZBTB10, IFITM1, GART, CDK19, PLEKHM3, RSF1, FN1, ZCCHC7, TGFBR3. The top down-regulated molecules were KRT1, CLCA2, ARTN, TPM4, VPS53, S100A8, ACOT11, HDHD2, FKBP5, GNG8. The upstream regulators inhibited in the BCNS keratinocytes samples were microRNAs miR-1-3p (no activation score), miR-381-3p, miR-19b-3p, miR-19b-3p, miR-374b-5p, miR-340-5p. JNK signaling and miR-101-3p regulator network was predicted to be associated with the signaling response of BCNS keratinocytes to rapamycin.

Similar analysis of fibroblast samples that was based on 3496 differentially expressed genes common to both normal and BCNS fibroblasts resulted in enrichment of canonical pathways involving cell cycle control of chromosomal replication, role of BRCA1 in DNA damage response, mitotic roles of Polo-like kinase, hereditary breast cancer signaling and role of CHK proteins in Cell cycle checkpoint control. The top up-regulated molecules are ADH1B, ITGB8, DIO2, EFEMP1, GRIA1, SLC40A1, RGCC, FMO2, SVEP1, CCL2, GLDN, RGCC, STEAP4, DAPK1. The top down-regulated molecules were ANLN, PBK, DLGAP5, RRM2, HMMR, TOP2A, SHCBP1, CEP55, TTK, NDC80, KIF20A, CCNB1, BIRC5. In both normal and BCNS fibroblasts samples, upstream regulators CDKN1A and TP53 were activated whereas the E2F1 and RABL6 were predicted to be inhibited in response to rapamycin. It is of interest to note that RABL6 plays a role in cell growth and survival and its overexpression is associated with tumorigenesis.

The canonical pathways enriched in the normal fibroblast samples, based on 1301 unique genes, included CHK proteins in cell cycle checkpoint control, neuregulin signaling, PPAR/RXR activation, role of BRCA1 in DNA damage response and VEGF signaling. The top up-regulated molecules were FGF7, SPIDR, SFRP2, SYNPO2, FMO3, FAM198B, WISP1, IL6, CEBPD, JAM2. The top down-regulated molecules were HAS2, TMPO, C4orf46, USP1, MIS18BP1, HMGB2, NETO2, PSAT1, BLM, TRIM59. In the normal fibroblasts samples, upstream regulators miR-495-3p, miR-381-3p, miR-124-3p, miR-590-3p, miR-340-5p were predicted to be active.

Based on 1463 unique genes differentially regulated in the BCNS fibroblasts only, mitochondrial dysfunction, oxidative phosphorylation, agrin interactions at neuromuscular junction, regulation of actin-based motility by Rho and estrogen receptor signaling were enriched canonical pathways. The top up-regulated molecules were TXNIP, FGF9, WISP2, TMTC1, ZBTB16, NEAT1, SEMA5A, IGF2, LRRN4CL, KCND3. The top down-regulated molecules were THBD, PHLDA1, TJP2, PRPS1, SRSF3, C4orf32, MATN2, ATOH8, EGR1, MLLT11. In the BCNS fibroblasts samples, the upstream regulators RICTOR and miR-22-3p ((miRNAs w/seed AGCUGCC) were activated. The upstream regulator 1,2-dithiol-3-thione was predicted to be inhibited whereas no prediction for HNF4A and miR-9-5p (and other miRNAs w/seed CUUUGGU). The top regulator effects networks were associated with FLI1, MMP3, CD3 and NFE2L2. These analyses showed involvement of different focus molecules in different cell type that contribute to the same canonical pathways hinting to common signaling pathways (Supplementay File 5).

### Networks enriched in BCNS samples after rapamycin treatment

**Figure 6:**
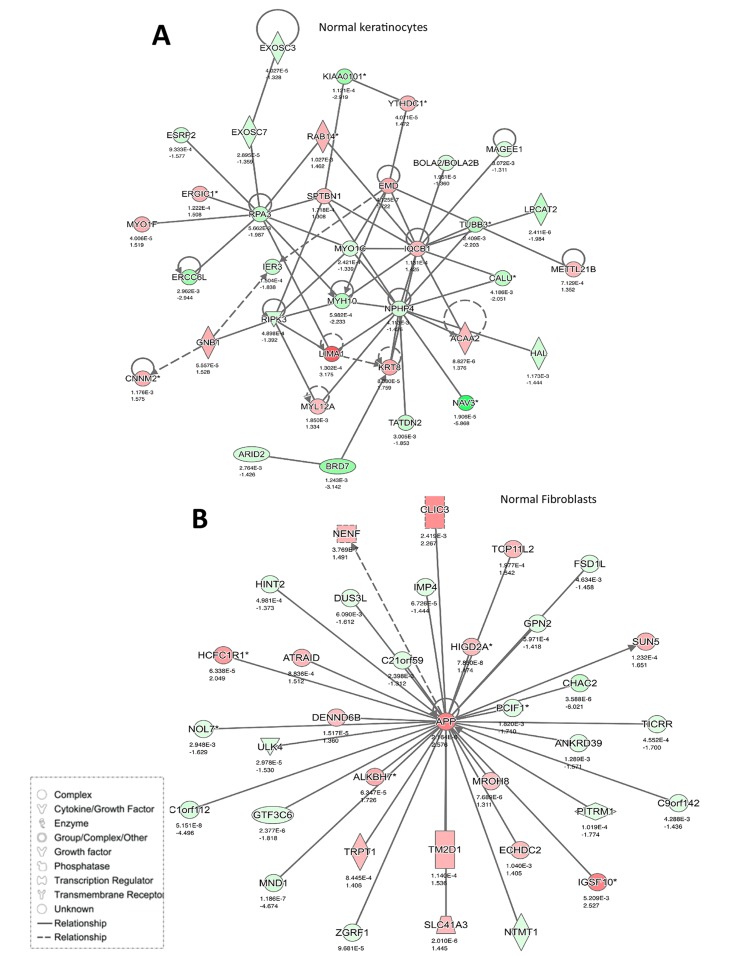
Networks enriched in normal cells after rapamycin treatment **(A)** Keratinocytes and **(B)** fibroblasts. Gene expression variation by at least 2-fold is depicted by color (red, up-regulated; green, down-regulated; gray, no significant change).

**Figure 7:**
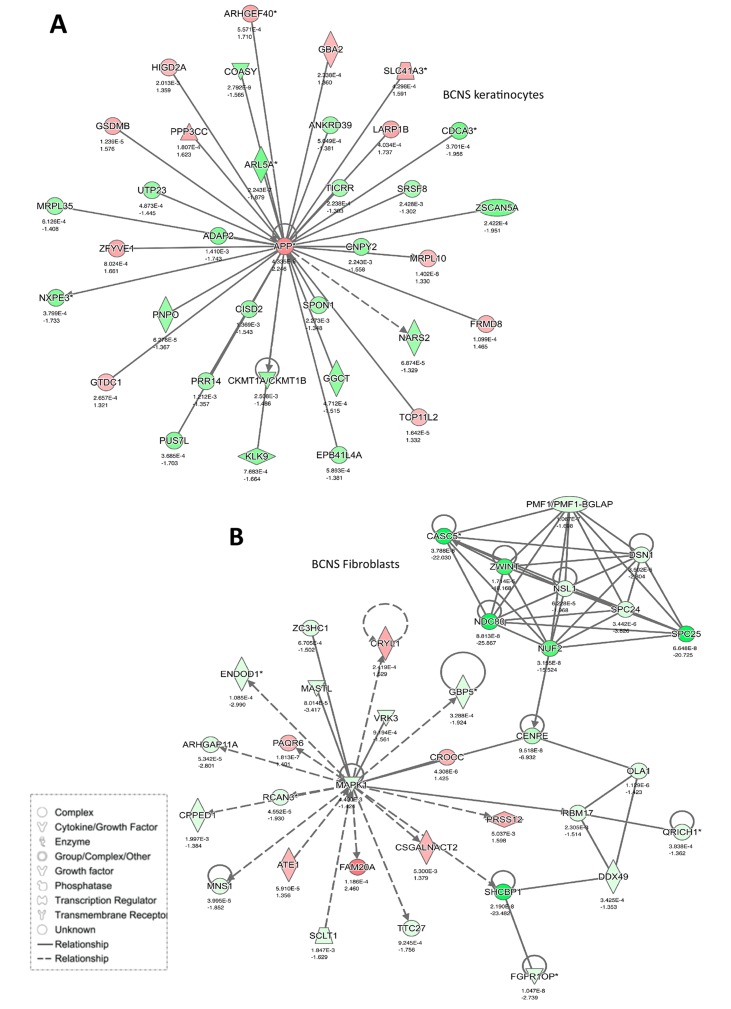
Networks enriched in BCNS cells after rapamycin treatment **(A)** Keratinocytes and **(B)** fibroblasts. Gene expression variation by at least 2-fold is depicted by color (red, up-regulated; green, down-regulated; gray, no significant change).

Here we performed an IPA network analysis that revealed several important such networks that are significantly altered post-rapamycin treatment (Figures 6 and 7). The most notably altered pathway in BCNS keratinocytes compared with normal keratinocytes, based on 35 focus molecules (score 50), was linked to digestive system development and function (Figure 6A and 7A). Several genes such as APP, SPON1 (cell adhesion promotes neurite growth and axonal guidance), CISD2 (associated with neurodegenerative Wolfram syndrome 2), KLK9 (associated with collagen biosynthesis and modification), CDCA3 (involved in cell cycle regulation mediated by APC) were included in this network and are related to development, neuronal growth and function.

Similar analyses of BCNS and normal fibroblasts, based on 35 focus molecules, showed enrichment of networks related to cell cycle, DNA replication, recombination and repair (Figure 6B and 7B). Notably, CASC5 (kinetochore-microtubule attachment and chromosome segregation, MAPK1 (involved in proliferation, differentiation, transcriptional regulation), and CROCC (centrosome cohesion before mitosis, associated with medulloblastoma) were included in this network.

## DISCUSSION

The cellular mechanisms whereby hedgehog (HH) pathway activation leads to BCCs are yet to be established [29]. Excess HH signaling may directly cause proliferation of BCC precursors [9, 52]. Alternatively, the HH pathway may be involved primarily in stem cell maintenance as has been shown in tumor stem cells for some tissues [8, 53, 54]. As either a direct effector of proliferation or maintainer of stem cells, HH would be acting in a cell autonomous role in BCCs. An alternative model involves a paracrine function in which HH signaling acts upon stroma to generate a tumor-promoting microenvironment while having a modest role in the tumor cells themselves [44, 45]. This model is appealing in the case of BCCs because of evidence that conditioned stroma is necessary for maintenance of tumor viability and growth. There are data supporting all three of these biological roles in cancer, and it is not clear which may apply to epidermal cell transformation [29]. Likewise, the fine details of the molecular mechanisms by which HH pathway activation exerts its effects are not entirely understood. Most investigations have focused on downstream targets of the GLI transactivators. A number of specific candidate genes have been studied in human and mouse BCCs including PDGF receptor, BCL2, cyclin D1 and D2. However, unbiased attempts by global gene expression to determine key HH target genes important in BCCs have yielded inconsistent results (reviewed in [22]).

Using exploratory analysis at baseline, we were unable to identify gene expression changes that molecularly distinguish affected individuals from controls. The lack of clear delineation of BCNS subjects from normal controls is depicted by PCA and HC analyses of fibroblasts or keratinocytes (Supplementary Figure 1 and Figure 1). Our results showing lack of BCNS sample clustering on the basis of mutation type or gender are in line with previous observations demonstrating lack of evidence for genotype-phenotype correlation in BCNS cases, including no association between mutation type, age of onset, or number of BCCs developed in BCNS patients [55]. Conceivably, subtle gene expression changes caused by single copy loss of *PTCH1* may enable the intact *PTCH1* gene to exert negative feedback control, particularly under *in vitro* growth conditions. Along these lines, previous studies showed that the up-regulated HH-signaling *in vivo* was suppressed in BCC and medulloblastoma (MB) cells grown *in vitro* [46, 47]. Moreover, HH signaling of MB cells grown *in vitro* was not restored upon transplantation in nude mice, nor did they respond to treatment with HH antagonists [47]. It is also interesting to consider whether apart from the classical tumor suppressor ‘two-hit’ model, the ‘continuum model’ [56] that accounts for subtle dosage effects, ‘obligate haploinsufficiency’, is associated with the effect of *PTCH1* tumor suppressor gene in BCNS.

Hence, our results at low stringency (p-value <0.05, no FDR cut-off; FC 1.3) have enabled us to unmask candidate baseline differences between BCNS and normal control cells that are likely to play a role in BCC growth under conditions in which the HH pathway has been suppressed [47]. Thus, a total of 585 and 857 genes differentially expressed in keratinocytes and fibroblasts samples from BCNS subjects, respectively were identified. For example, a Gene Set Enrichment Analysis (GSEA) of highly regulated candidate genes recognized pancreatic β cell hallmark genes and mTOR signaling genes in BCNS keratinocytes, whereas analyses of BCNS fibroblasts candidate genes showed gene signatures that affect pathways regulating pluripotency of stem cells, including the WNT pathway. Moreover, these differentially affected gene signatures included canonical and non-canonical HH pathways in BCNS keratinocytes and fibroblasts that are likely to play a role in BCC development. One such example that is likely relevant in this context is our finding that MKL1 is up-regulated in BCNS keratinocytes, since its activation within the non-canonical HH pathway has recently been shown to promote drug resistance of BCCs [57].

Importantly, the phenotypic features of BCNS patients shown in our study and elsewhere [4, 58–61], provide strong support for a two-hit mechanism wherein the first hit is associated with early molecular changes and a variety of developmental defects occurring concurrently. While some of these defects may in fact arise through a two-hit mechanism, it is difficult to imagine how such symmetric features as generalized overgrowth, macrocephaly, coarse facies, and hypertelorism could arise through any mechanism other than one invoking an effect of haploinsufficiency. Hence, gene expression patterns in tissues at some stages of development must almost certainly differ between BCNS subjects and controls. Accordingly, networks of differentially regulated genes at baseline shown here are of interest as they were found to be enriched in processes which might play important roles in the development of congenital phenotype (e.g., macrocephaly, skeletal abnormalities, bossing of forehead). For example, BEX1 (Brain Expressed, X-Linked 1) plays a role in cell cycle progression and neuronal differentiation, and is related to p75NTR/NGFR-mediated signaling and it inhibits neuronal differentiation in response to nerve growth factor (NGF). SOX4 (SRY-related HMG-box) is a member of SOX family of transcription factors that regulate pathways in embryonic development and cell fate determination and is related to ERK signaling. MEG3 (Maternally Expressed 3) may function as a lncRNA tumor suppressor as it inhibit tumor cell proliferation, interacts and regulates p53 gene expression and its down-regulation has been experimentally observed in cancer cell lines of various origins. SYN1 (Synapsin 1) is member of neuronal phosphoproteins that function to regulate axonogenesis and synaptogenesis. SIM2 (Drosophila single-minded gene homolog) encodes for transcription factor that regulates neurogenesis and may have pleiotropic effects in tissues during development. Thus, perturbations caused by loss of function *PTCH1* mutations although subtle, seem sufficient to result in developmental anomalies and BCCs in BCNS patients. The occurrence of *PTCH1* mutations that result in developmental anomalies concomitant with BCC expression serve as poignant reminder of the role of *PTCH* in normal tissue development and as a tumor suppressor gene (TSG), a basic tenet in cancer biology, including all TSGs identified to date [62].

Prior studies suggest that signaling through the hedgehog and mTOR pathways have separate, albeit synergistic effects on malignant transformation [33–35, 37]. Indeed, our present GSEA studies (vide supra) showed an activated mTOR pathway in BCNS *PTCH1* (+/-) keratinocytes. Accordingly, mTOR/S6K1 and HH pathways coincidentally target an overlapping set of genes that are key modulators of HH-related carcinogenesis [38–40]. In this regard, a recent study has demonstrated the inhibition of human rhabdomyosarcoma xenografts by rapamycin through its effect on HH effector genes such as Gli1 and Gli 2 as well as *PTCH1* and *PATCH2* [63]. These effects were associated with over 80% reduction in cyclin D1, a downstream transcription target for both HH and mTOR signaling pathways [63].

To investigate the differential effect of rapamycin in BCNS, we compared the gene expression profiles of keratinocytes and fibroblasts derived from normal subjects and from unaffected skin cells of BCNS patients. Accordingly, rapamycin affected the expression of 1411 genes and 3214 genes which were altered in BCNS and normal keratinocytes, respectively (FC >1.3 and <-1.3; FDR <0.05), including 812 genes whose altered expression was shared by both group. A similar analysis of BCNS and normal fibroblasts resulted in 4959 genes and 4797 genes, respectively wherein expression in each group was altered following rapamycin treatment, including expression of 3496 genes that was common in both groups. The increased stringency in analyzing the rapamycin effect as compared with baseline's lesser stringency was meant to focus on the most salient alterations seen due to rapamycin. We focused on these gene lists via an IPA analysis in order to discern differences in molecular functions and canonical signaling. For example, our results show that rapamycin treatment of BCNS keratinocytes affected Wnt/β-catenin Signaling, ILK signaling, epithelial adherens junction signaling, Sonic Hedgehog signaling, IGF-1 signaling, caveolar-mediated endocytosis signaling, and amyloid processing (Supplementary Data). On the other hand, pathways that were associated with response to rapamycin in BCNS fibroblasts include paxillin signaling, DNA damage-induced 13-3-3σ, integrin signaling, Cdc42 signaling, telomere extension by telomerase, HGF and VEGF signaling. The overlap between hedgehog targets and rapamycin targets provides evidence for a model in which the anti-neoplastic effect of rapamycin relates to suppression of HH signaling. Rapamycin is the subject of approximately 1500 clinical trials for cancer treatment and prevention listed on the NIH Clinical Trials web site (clinicaltrials.gov). Among these trials, five ongoing or recently completed studies relate to the use of this compound in prevention of skin cancer in transplant recipients.

This study underscores the need to catalog baseline molecular differences in BCNS cohorts in order to anticipate the numerous manifestations characteristic of BCNS, particularly the locally invasive but seldom metastatic nature of BCCs. Perturbations caused by loss of function of *PTCH1* mutations unmasked herein, including alterations in canonical and noncanonical HH pathways, although subtle, seem sufficient to result in developmental anomalies and BCCs in BCNS patients. This is the first study involving skin derived keratinocytes and fibroblasts from BCNS patients wherein global gene profiling may be used to generate rationale hypotheses and design of future experiments, including noncanonical HH pathways.

## MATERIALS AND METHODS

### Ethics statement

The clinical protocol under which this study (https://clinicaltrials.gov/ct2/show/record/NCT00433485) was conducted was approved by Yale University's Institutional Review Board, the Clinical Trials Protocol Review Committee of the Yale Comprehensive Cancer Center, and by the sponsoring agency, the Division of Cancer Prevention, National Cancer Institute. Informed consent was obtained to collect tissue from study participants meeting clinical diagnostic criteria for familial BCNS and normal individuals in accordance to the ethical standards and principles expressed in the Declaration of Helsinki.

### Study participants

Four female and five male BCNS cases were ascertained through the clinical practices and previous research programs (AEB and DJL). Eight age- and sex- matched, unaffected controls included relatives of cases and normal volunteers at Yale University. The mean ages of cases and controls were virtually identical - 42.3+/-14.0 and 41.6+/-12.5 - although female cases were older on average than female controls while male cases were younger on average than male controls (Table 1).

Cases were chosen on the basis of meeting clinical criteria for BCNS [4]. In addition, *PTCH* gene sequencing was performed by the CLIA-certified DNA Diagnostics Laboratory in the Department of Genetics at Yale. Four affected subjects had a mutation predicted to truncate the PTCH protein. Three had missense alterations in codons conserved from humans to invertebrates and not seen in approximately 125,000 normal controls (gnomAD browser: http://gnomad.broadinstitute.org/gene/ENSG00000185920). One of these mutations was reported previously in BCNS [40]. Subject number 18 had no clearly deleterious mutation. However, the diagnostics methods used in this study would not have detected exon-size or larger deletions and duplications or other large-scale rearrangements, such as inversions. This subject was heterozygous for SNPs throughout the gene, ruling out “hemizygosity” (heterozygous deletion) of the whole gene. Subject 22 had an alteration in exon 1E predicted to create a highly efficient, abnormal splice acceptor site (http://www.fruitfly.org/seq_tools/splice.html). This variant was not seen in 125,000 normal controls. Among the controls, four were relatives of cases involved in this study, and all tested negative for the mutation found in their affected relative. Among the remaining four controls, none had any of the exclusion criteria listed below:

- One or more basal cell carcinomas
- Palmar or plantar pits typical of BCNS
- History of medulloblastoma
- History of odontogenic keratocyst or any jaw cyst for which a histopathologic diagnosis could not be ascertained
- History of ovarian or cardiac fibroma
- Polydactyly
- Macrocephaly determined after adjustment for height
- Craniofacial features of BCNS including cleft palate, frontal bossing, hypertelorism, iris coloboma or other developmental defects of the eye, or other typical facial features.

### Specimen acquisition and tissue culture

Four 5 mm punch biopsies were obtained from unaffected skin of the upper inner arm of each case and control. Keratinocyte and fibroblast cultures were established as previously described [41]. Briefly, keratinocytes were harvested from epidermis by overnight exposure to Dispase at 4°C and seeded onto mitomycin C-treated feeders of 3T3 cells. Once colonies of keratinocytes were growing, they were expanded in low calcium MCDB medium. Fibroblasts were harvested from minced pieces of dermis obtained after dispase treatment of skin biopsies and expanded in DMEM plus 10% serum. Twenty-four vials of low-passage cells with approximately 5×10^5^ cells/vial were frozen in liquid nitrogen from each subject.

For experiments examining the effects of rapamycin, primary keratinocyte and fibroblast cultures were plated at approximately 25% confluence in 60 mm plates. One day after seeding, they were treated with 0μM (vehicle only), 10μM or 50μM rapamycin in DMSO (Sigma, Inc.) for 48 hours. Cells were nearly confluent when harvested.

### Gene expression and statistical analysis

#### RNA extraction

Total RNA was extracted from the keratinocyte and fibroblast cultures using the standard TRIzol^®^ (Invitrogen) protocol and was further purified using the RNeasy cleanup procedure (Qiagen) according to manufacturer's instructions. The quality of total RNA was assessed by Agilent 2100 Bioanalyzer (Agilent Technologies, Palo Alto, CA) for visual absence of genomic DNA contamination and integrity of 28S and 18S bands. Only samples with an A260/A280 ratio of at least 1.9 were used for microarray analysis. Samples were stored in 5μg aliquots at -70° C until use.

### Microarray processing and data analysis

Gene expression profiling was performed on total 102 samples on Affymetrix Human Genome U133 Plus 2.0 GeneChip Array^®^, comprising of fibroblasts and keratinocytes treated with vehicle, low- and high-dose rapamycin. Microarray processing was performed at Yale. The cDNA and cRNA preparation, cRNA labeling and hybridization to Affymetrix Human Genome U133 Plus 2.0 GeneChip^®^ microarrays (Affymetrix, Santa Clara, CA) was performed as per standard protocols according to manufacturer's instructions. The arrays were scanned using Hewlett-Packard GeneArray Scanner and the scanned output files were visually inspected for hybridization artifacts. The data quality was checked for the presence of spiked control cRNAs, background values caused by array autofluorescence and non-specific binding, and Q value. The microarray gene expression data have been deposited in Gene Expression Omnibus (GSE#120242).

The microarray data in.cel files was imported and RMA normalization performed using Partek^®^ Genomics Suite^®^ software, version 6.6^©^; 2014 (Partek Inc., St. Louis, MO, USA) followed by QC metrics and preliminary exploratory analysis. Attributes were assigned to the microarray files to the random effects namely subject and scan dates, and the fixed effects namely the cell type, gender, PTCH mutation type and rapamycin treatment followed by exploratory principal components analysis to observed clustering based on various sample attributes.

For unsupervised analysis, first probe level data was collapsed to gene level data by retaining for each gene, amongst multiple possible probes, the probe that had maximum coefficient of variation. All other probes were discarded. After collapsing the probe level data to gene level data, a secondary filter to exclude genes with coefficient of variation less than 0.1 (exclude genes with CV<0.1) was applied to normalized data resulting in 5384 and 3920 genes in keratinocytes and fibroblasts respectively that were clustered using Euclidean and average linkage method as similarity measure to identify patterns of clustering in the samples. Batch effect arising due to samples being run at different time points was studied using the statistical batch effect removal tool as per the manual (Partek GS's User Manual). To ensure that the batch effect is not the major source of variation, scan dates were included in the ANOVA model as well as sources of variation were studied. To identify differentially expressed genes, a mixed- model ANOVA with restricted maximum likelihood (REML) method to estimate variance components was used in this unbalanced, mixed model study that takes into account both ‘fixed’ experimental factors such as with disease status/mutation type, cell type, treatment and ‘random effects’ such as scan dates and subject. To generate gene lists for contrasts included in the ANOVA-REML design, significance of change cut-offs of FDR <0.05 and size of fold change >1.3 or <-1.3 were used.

### Gene Set Enrichment Analysis (GSEA)

GSEA was performed on the comprehensive microarray datasets for control and treatment comparisons to determine differences and enriched gene sets in the normal and BCNS groups. GSEA was performed as per software instruction to estimate the association between predefined gene sets in the MSigDb and the phenotype defined by the gene expression profiles of BCNS and normal keratinocytes and fibroblasts before and after treatment with rapamycin [64, 65]. The comparison of the groups was performed using GSEA by permuting the phenotype for 1000 times, and selecting the weighted scoring scheme with signal to noise statistical metric to rank genes and complete the GSEA analysis.

### Ingenuity Pathway Analysis (IPA^®^)

Following ANOVA, gene lists were created by applying false discovery rate (FDR), calculated using Benjamini's method [66] cut-off of <0.05 before uploading to IPA. For pathway analysis, these datasets with p-values and fold changes for each logical comparison were analyzed through QIAGEN's Ingenuity^®^ Pathway Analysis^®^ (IPA^®^, QIAGEN Redwood City, https://www.qiagen.com/ingenuity) using flexible file format, Affymetrix as identifier, and Human Genome U133 Plus 2.0 array as the platform used.

## SUPPLEMENTARY MATERIALS FIGURES AND TABLES













## Abbreviations

AGRN: agrin
BCNS: Basal Cell Nevus Syndrome
BCC: basal cell carcinoma
DEPTOR: DEP domain-containing mTOR-interacting protein
FDR: false discovery rate
FC: fold change
GSEA: gene set enrichment analysis
HC: hierarchy clustering
HH: hedgehog
HIP: hedgehog interacting protein
IPA: Ingenuity Pathway Analysis
mTOR: mammalian target of rapamycin
mLST8: mTOR Associated Protein
PI3K: Phosphoinositide 3-kinase
PTCH: Patched
PCA: principle component analysis
PRAS40: proline-rich Akt substrate of 40 kDa
RAPTOR: Regulatory-associated protein of mTOR
SMO: smoothened
TTi1-TEL2: Tel two interacting protein 1- telomere maintenance 2
