# Supplementary material for: Global gene expression of histologically normal primary skin cells from BCNS subjects reveals “single-hit” effects that are influenced by rapamycin
Source: Oncotarget. 2019 Feb 15;10(14):1360–87. doi: 10.18632/oncotarget.26640 (PMC6402716; doi:10.18632/oncotarget.26640)
Supplement: Supplementary file 1 [file oncotarget-10-1360-s001.pdf]

Global gene expression of histologically normal primary skin cells from BCNS subjects reveals "single-hit" effects that are influenced by rapamycin

SUPPLEMENTARY MATERIALS

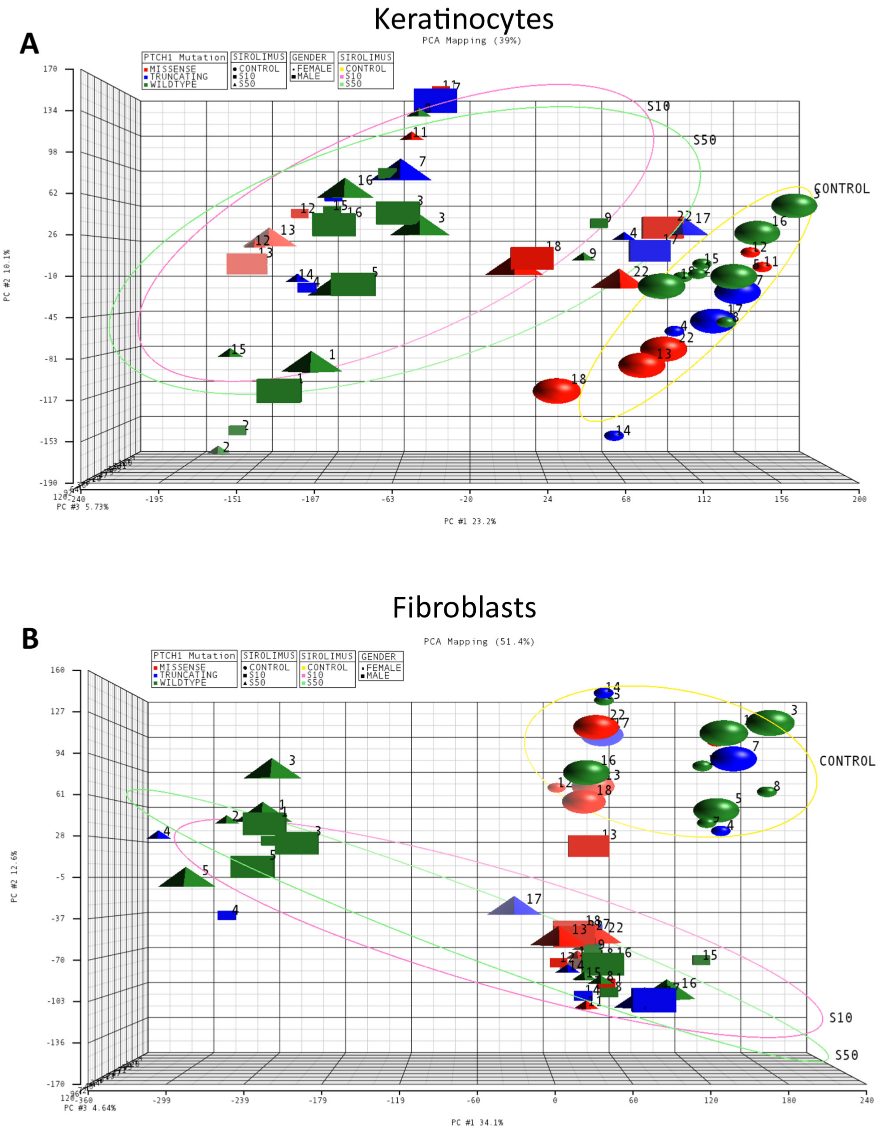

Supplementary Figure 1: Principal components analysis (PCA) comparing BCNS subjects to normal subjects. Each dot represents one of the 51 samples profiled using HG_U133 Plus.2.0 microarray chip which are colored according to PTCH1 mutation status (Green, WT; Red, Missense; Blue, Truncating); each shape represents the treatment (sphere, Control; Cube, sirolimus/rapamycin low dose (S10); tetrahedron, sirolimus/rapamycin high dose (S50)) and size represents the gender of study participants (small shapes, female; large shapes, male). The ellipses drawn are used to visually assess similarities and how samples are grouped together (Yellow, Control; Pink, sirolimus low dose (S10); Green, sirolimus high dose (S50)). PCA identified three directions shown in 3D PCA plot that depicts maximum variation along PC#1 (23.2%), followed by PC#2 (10.1%), and PC#3 (23.2%) along X, Y and Z axis (PCA mapping of 39%) in keratinocytes **(A)** and maximum variation along PC#1 (34.1%), followed by PC#2 (12.6%), and PC#3 (4.64%) along X, Y and Z axis (PCA mapping of 51.4%) in fibroblasts **(B)**.

Supplementary Data 1: ANOVA design used for study.

See Supplementary File 1

Supplementary Data 2: GSEA analysis results.

See Supplementary File 2

Supplementary Data 3: GSEA NES, nom-p-value and q-values.

See Supplementary File 3

Supplementary Data 4: Normalized counts of array data.

See Supplementary File 4

Supplementary Data 5: IPA core analysis results.

See Supplementary File 5
